# Bridging waste-derived carbon catalyst innovation and machine learning for sustainable biodiesel production: a comprehensive review

**DOI:** 10.1039/d6ra05871j

**Published:** 2026-10-19

**Authors:** Menna Ebrahim, Mostafa Mahmoud, Fatma H. Ashour, Osama A. Ibrahim, Nourhan H. Khashaba

**Affiliations:** a Chemical Engineering Department, Faculty of Engineering, Cairo University Giza 12613 Egypt menna.202211310@eng-st.cu.edu.eg; b Public Works Department, Faculty of Engineering, Cairo University Giza 12613 Egypt

## Abstract

Biodiesel is a promising sustainable alternative to fossil diesel, but its deployment is limited by challenges in catalyst efficiency, stability, reusability, and process optimization. Metal-doped activated carbon (AC) and biochar-based catalysts have attracted considerable attention owing to their high surface area, tunable porosity, and low-cost production from waste biomass. In parallel, machine learning (ML) has emerged as a powerful tool for biodiesel yield prediction, process modeling, and optimization. However, these research areas have largely progressed separately. This review combines scientometric analysis with a systematic review to examine advances in waste-derived AC and biochar-based catalysts and ML applications in biodiesel production. Catalyst synthesis, activation, metal doping, physicochemical properties, catalytic performance, stability, and reusability are critically evaluated alongside ML-based prediction and process optimization approaches. An integrated catalyst–process–performance framework is further discussed to highlight opportunities for incorporating catalyst descriptors into data-driven biodiesel modeling. The review identifies key research trends, knowledge gaps, and future opportunities for advancing sustainable and scalable biodiesel production.

## Abstract 2

This review integrates waste-derived metal-doped activated carbon and biochar catalysts with machine learning to advance biodiesel yield prediction, process optimization, catalyst stability, and sustainable production.

## Graphical abstract

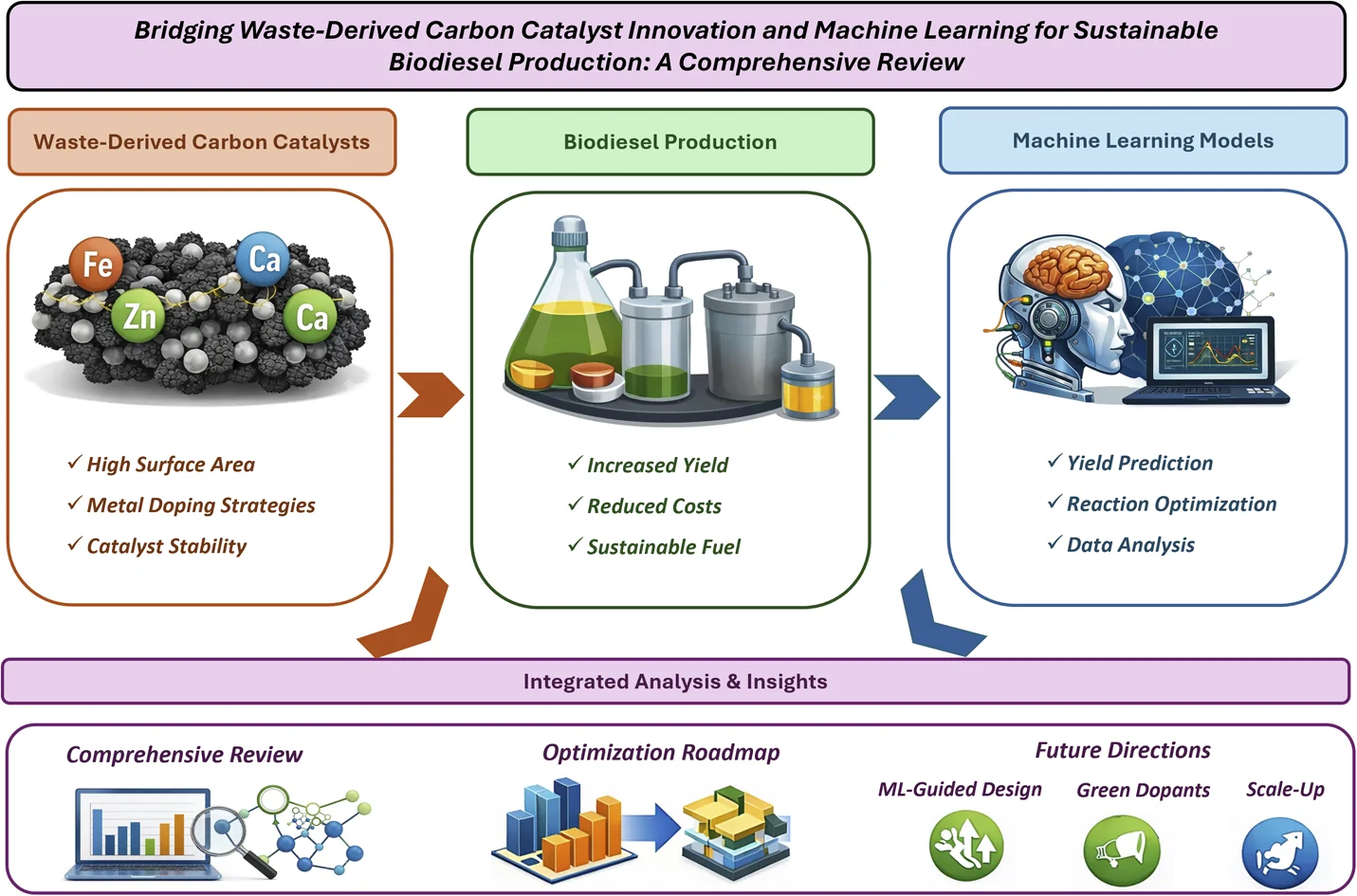

## 1. Introduction

### 1.1 Background

Biodiesel is a renewable, oxygenated alternative to petroleum diesel, typically composed of fatty acid methyl esters (FAMEs) and produced primarily by the transesterification of biological lipid feedstocks such as waste cooking oil (WCO), vegetable oils, animal fats, and microalgae.^1^ Feedstock choice strongly influences production cost, fuel quality, and process requirements. Edible oils provide high conversion and favorable fuel properties, but raise food security and land-use concerns. These issues motivate the use of non-edible, waste-derived, and algal feedstocks, which reduce competition with food supplies but introduce greater compositional variability and higher free fatty acid (FFA) levels that often require pretreatment.^2^ Key process variables (including the methanol-to-oil ratio, temperature, catalyst type and loading, and reaction time), along with feedstock FFA and moisture levels, determine conversion, selectivity, and downstream purification needs.^3^

Among production routes, catalytic transesterification remains the dominant industrial pathway because of its balance of simplicity, mild conditions, and high conversion potential; nevertheless, alternative approaches (including supercritical methanol, microemulsions, and process intensification *via* microwave or ultrasonication) have demonstrated advantages for specific feedstocks or process goals.^4^ Catalyst choice is central to process performance. Homogeneous basic catalysts (*e.g.*, NaOH and KOH) provide rapid kinetics but face challenges such as difficult separation, glycerol contamination, and low recyclability. In contrast, heterogeneous and nanostructured catalysts offer easier separation, better reusability, and lower environmental impact, although they can be more expensive and may exhibit mass-transfer limitations.^5^ Recent studies show that carefully engineered heterogeneous systems, including waste-derived AC and metal-doped biochar/AC supports, can achieve high yields (>90%) under mild conditions and introduce desirable functionalities such as magnetic separation or bifunctional acidic/basic sites.^6,7^

AC is a particularly attractive support due to its high surface area, tunable porosity, and surface chemistry, enabling dispersion and stabilization of active metal species and the tailoring of acid/base character for both esterification and transesterification reactions.^8^ Metal-doped AC and biochar catalysts (*e.g.*, CaO, Fe, Ni, and Mn) can enhance active site density, mass transfer, and magnetic recoverability, thereby potentially improving catalytic performance and reusability. Challenges such as metal leaching, pore blockage, and synthesis complexity can be minimized through controlled metal loading, strong metal–support anchoring, and surface stabilization, which can enhance catalyst durability and biodiesel fuel purity.^3,9^

Modeling of biodiesel production and quality has traditionally relied on mechanistic and semi-empirical approaches, including kinetic models (*e.g.*, pseudo-homogeneous and Langmuir–Hinshelwood–Hougen–Watson formulations), thermodynamic phase-equilibrium models, and response surface methodology (RSM). While these conventional models provide valuable physical insight, they often require extensive experimental calibration, simplifying assumptions, and precise knowledge of reaction mechanisms and catalyst behavior. Their applicability is further constrained when dealing with complex feedstocks, heterogeneous catalysts, multi-phase systems, and strongly nonlinear interactions among operating parameters. As a result, conventional models may struggle to generalize across different feedstocks and operating conditions, motivating the growing adoption of data-driven modeling approaches.^2,10^

Parallel to materials development, machine learning approaches have matured into powerful tools for modeling, optimization, and process control in biodiesel research. Supervised learning models rely on labeled experimental data to establish relationships between process variables (*e.g.*, catalyst loading, temperature, and alcohol-to-oil ratio) and output responses such as biodiesel yield and fuel properties. Commonly used supervised models include artificial neural networks (ANNs), particularly multilayer perceptrons (MLPs), support vector machines (SVMs), and random forests (RF). Hybrid approaches combine ML with optimization or statistical methods, including genetic algorithms, to improve prediction and process performance, such as through ANN-GA and RSM-GA models.^11,12^

In addition, ensemble techniques, which integrate predictions from multiple base learners, have demonstrated robust performance by improving generalization and reducing overfitting. Collectively, these ML approaches have shown excellent predictive capability for biodiesel yield and key physicochemical fuel properties, including cetane number (CN), viscosity, density, and cold-flow indices, while significantly reducing experimental time and cost.^11,12^ Emerging physics-informed and hybrid ML methods offer additional promise by embedding mechanistic constraints into learning, helping to bridge the gap between limited experimental datasets and physically consistent predictions.^13^ Although many ML studies rely on small, feedstock-specific datasets and use various evaluation metrics, these models effectively capture complex nonlinear relationships and enable accurate prediction and optimization, underscoring their importance for advancing biodiesel catalyst and process development.^14^

### 1.2 Research gaps

Despite substantial advances in waste-derived and metal-doped carbon catalysts and in machine-learning-based biodiesel modeling, these research areas have largely progressed independently. As summarized in Table 1, existing reviews predominantly address catalyst development, reactor/process engineering, or ML modeling separately, with limited consideration of their interrelationships. In particular, limited attention has been given to how catalyst synthesis and physicochemical characteristics can be considered alongside feedstock properties and reaction conditions in data-driven biodiesel modeling. Thus, an integrated synthesis of catalyst development, catalytic performance, and ML-assisted biodiesel optimization remains insufficiently developed. This gap highlights the need for a systematic review that not only evaluates these research streams individually but also examines opportunities for their integration.

**Table 1:** A comparative overview of existing reviews in biodiesel production systems and ML integration

Study	Study concerns and contributions							
	Methodological approach	Catalyst coverage			ML	Catalyst performance evaluation	Sustainability perspective	Scientometric analysis
		AC	Biochar	Metal doping				
4	Catalyst review	✗	✗	✓	✗	✓	✓	✗
6	Reactor engineering review	✗	✗	✗	✗	✓	✓	✗
15	Nanocatalyst review	✗	✗	✓	✗	✓	✓	✗
16	Heterogeneous catalysis review	✗	✗	✓	✗	✓	✓	✗
17	Carbon catalyst review	✗	✗	✓	✗	✓	✓	✗
18	Waste-derived catalyst review	✓	✓	✗	✗	✓	✓	✗
19	Functional carbon catalyst review	✓	✓	✗	✗	✓	✓	✗
20	Biochar catalyst review	✗	✓	✗	✗	✓	✓	✗
21	ML/AI modeling review	✗	✗	✗	✓	✗	✓	✗
22	Reactor configuration review	✗	✗	✗	✗	✓	✓	✗
23	Data-driven modeling review	✗	✗	✗	✓	✓	✓	✗
24	ANN mini review	✗	✗	✗	✓	✗	✓	✗
25	ANN critical review	✗	✗	✗	✓	✓	✓	✗
26	ML algorithm comparison review	✗	✗	✗	✓	✓	✓	✗
Proposed study	Integrated catalyst–ML review	✓	✓	✓	✓	✓	✓	✓

### 1.3 Research contributions

The main aim of this review is to critically connect waste-derived activated-carbon and biochar catalyst engineering with catalyst-aware machine learning for biodiesel production. Specifically, the review examines how catalyst composition and physicochemical properties, feedstock characteristics, and reaction conditions can be integrated within a structure–property–process–performance framework to support catalyst screening, biodiesel prediction, and process optimization.

To achieve this aim, the review combines scientometric mapping with systematic analysis to examine the evolution of both catalyst development and computational approaches and to establish an analytical basis for integrating these research streams. The main contributions of this manuscript are as follows:

- Combining scientometric analysis and systematic review to capture research evolution, major research themes, and current developments in waste-derived carbon catalysts and ML-based biodiesel production.

- Systematically evaluating synthesis, activation, functionalization, and metal-doping strategies for activated carbon and biochar catalysts derived from agricultural and cellulosic wastes.

- Critically comparing representative catalytic systems in terms of catalytic activity, stability, reusability, and practical limitations relevant to biodiesel production.

- Assessing the development of ML applications for biodiesel yield prediction, reaction modeling, process optimization, catalyst-related analysis, and scale-up.

- Examining opportunities for catalyst-aware ML integration by relating catalyst synthesis and physicochemical descriptors to feedstock characteristics, reaction conditions, catalytic performance, and multi-objective optimization.

- Identifying research gaps and future directions for developing standardized catalyst–process–performance datasets and experimentally validated data-driven approaches for sustainable biodiesel production.

By bringing these research streams together, the review consolidates fragmented knowledge and highlights opportunities for linking catalyst engineering with data-driven methodologies to improve biodiesel efficiency, stability, reusability, scalability, and process optimization.

The remainder of this paper presents the methodology, scientometric results, systematic review of metal-doped AC and biochar catalysts, and parallel review of ML applications in biodiesel prediction and optimization. It concludes with current limitations, research gaps, and future directions for integrating catalyst design with advanced ML approaches.

## 2. Methodology

### 2.1 Hybrid review approach

A hybrid review approach was employed to organize and evaluate research on metal-doped AC catalysts for biodiesel production. As shown in Fig. 1, this approach is based on two complementary methods: quantitative scientometric analysis, which examines publication data, citation patterns, and research metrics to reveal scientific trends, and a qualitative systematic review, which provides a structured and transparent evaluation of research findings.

**Fig. 1:**
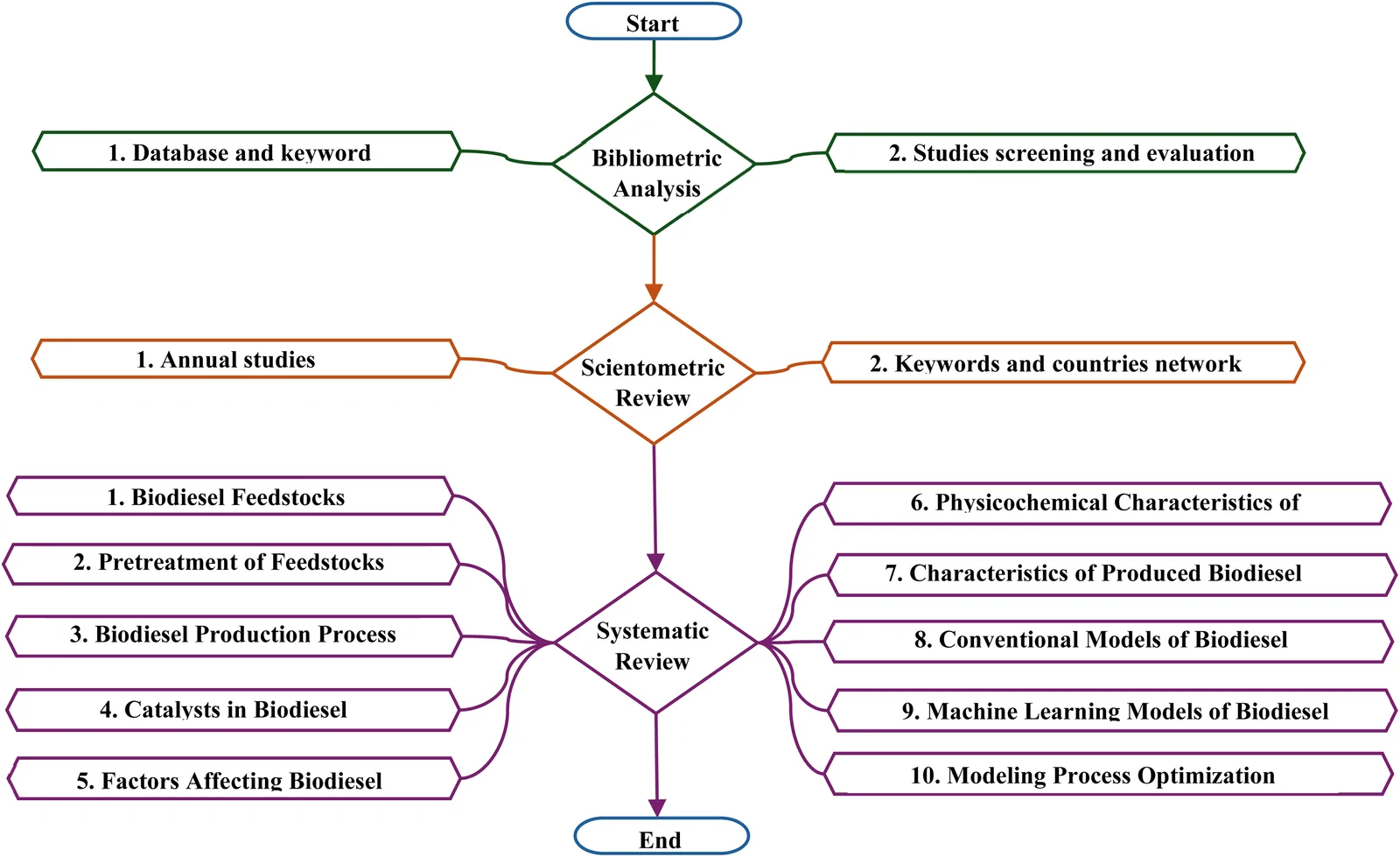
Overview of the integrated review framework.

Together, these two complementary review methods form the analytical framework that integrates catalyst development and machine learning–based biodiesel optimization within a unified perspective.

### 2.2 Bibliometric analysis

The research process began by selecting databases and keywords to gather studies on metal-doped AC catalysts for biodiesel production. Retrieved publications were filtered using strict criteria, and the final set was evaluated for quality and relevance as illustrated below.

#### 2.2.1 Database and keyword selection

The primary database used to identify relevant publications was Scopus, selected because of its broad coverage of peer-reviewed research in chemical engineering, catalysis, renewable energy, biofuels, and related fields. The literature search was conducted using the TITLE-ABS-KEY field to identify relevant studies from titles, abstracts, and author keywords. The search strategy was designed to provide broad coverage of biodiesel production, waste-derived activated carbon and biochar catalysts, catalyst modification, and machine-learning-based analysis and optimization. The detailed search strategy and study selection criteria are provided in Table 2.

**Table 2:** Literature search strategy and study selection criteria

Component		Details
Database		Scopus
Search strategy		Biodiesel AND activated carbon AND metal-doped catalyst-related terms, combined with catalyst, biochar, carbon, biodiesel production, process, and ML-related terms using boolean operators
Search field		TITLE-ABS-KEY (title, abstract, and author keywords)
Exact boolean expression		TITLE-ABS-KEY ((“biodiesel”) AND (“activated carbon” OR “biochar” OR “waste-derived carbon”) AND (“catalyst” OR “metal-doped” OR “metal doping” OR “heterogeneous catalyst” OR “transesterification” OR “esterification” OR “biodiesel production” OR “machine learning” OR “artificial intelligence” OR “optimization”))
Criteria	Inclusion	Peer-reviewed journal articles; full-text available; published up to 2025, relevant to biodiesel production and waste-derived carbon/activated-carbon catalysts, catalyst modification/metal doping, and/or ML-based biodiesel analysis
	Exclusion	Conference proceedings; books/book chapters; non-English publications; studies outside the defined research scope; duplicate records

The retrieved records were screened according to the predefined inclusion and exclusion criteria. The primary systematic and scientometric dataset was established from the February 2026 search. A targeted revision-stage literature update was subsequently conducted to capture relevant recent studies, including 2026 publications, which were used only to update the narrative discussion and not the original bibliometric dataset. This distinction preserved the consistency and reproducibility of the primary dataset.

#### 2.2.2 Studies screening and evaluation

The Preferred Reporting Items for Systematic Reviews and Meta-Analyses (PRISMA) workflow was applied to ensure a transparent, structured, and reproducible study-selection process, as shown in Fig. 2. Following the Scopus search, the retrieved records were downloaded and processed before screening. Duplicate records were identified and removed prior to title and abstract screening. Records were then screened based on their titles and abstracts, followed by full-text assessment of potentially relevant studies. After duplicate removal and application of the initial exclusion criteria, 423 articles remained. Title and abstract screening reduced the dataset to 288 studies, while full-text evaluation further narrowed the selection to 119 papers. The detailed screening and selection process is presented in Fig. 2.

**Fig. 2:**
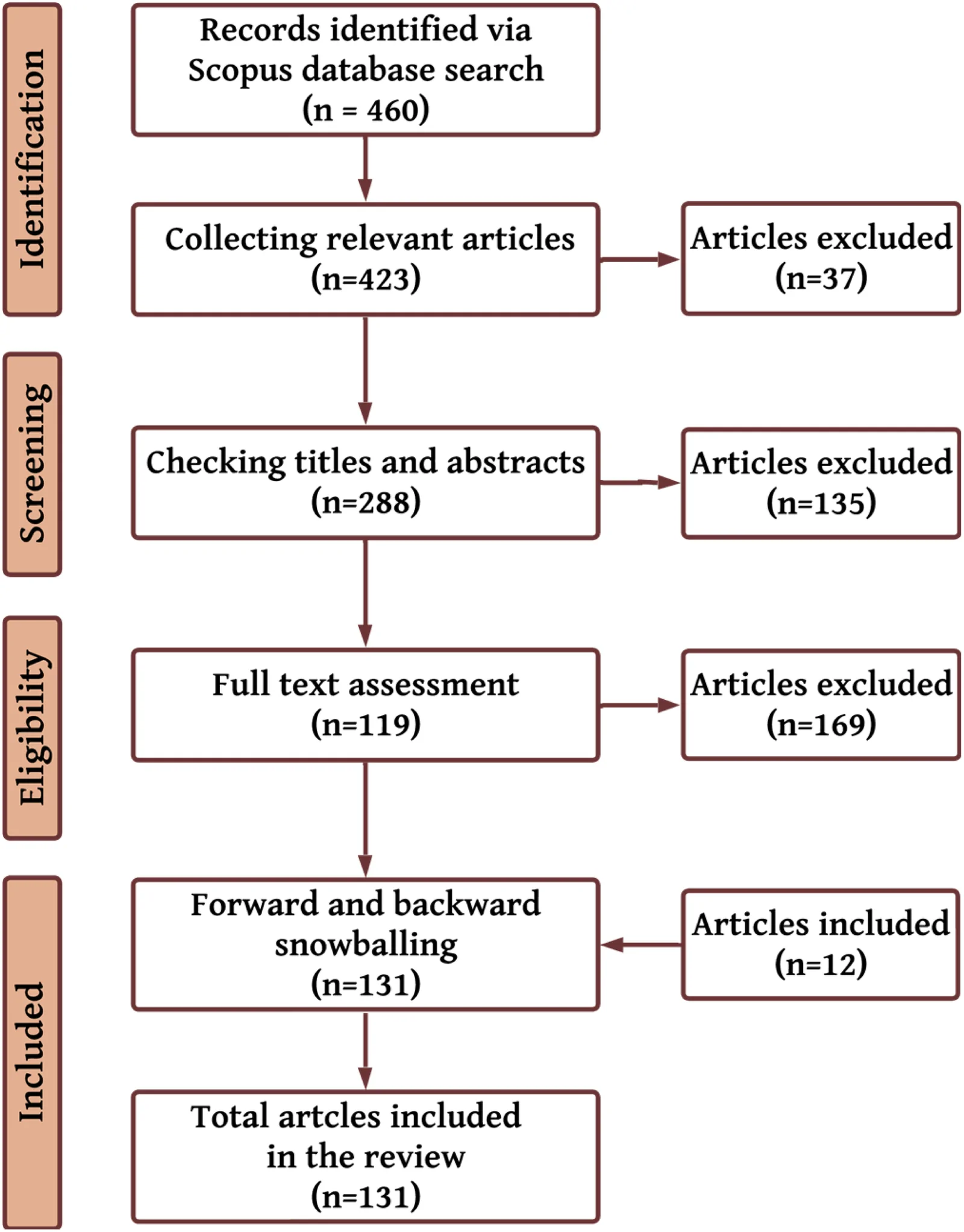
PRISMA-based schematic of the literature search and filtering procedure.

To enhance the coverage of the literature, snowballing was subsequently performed by examining the reference lists and citation records of the selected studies. This process identified additional relevant publications that were not captured by the initial Scopus search. Following this additional screening, the total number of reviewed studies increased to 131. The final set of 131 studies was used for both the scientometric and systematic review analyses.

## 3. Scientometric review results

The first review type of the proposed analytical framework, scientometric analysis, examines publication trends, keyword patterns, and international collaboration networks, as discussed below.

### 3.1 Annual studies progress

Publication output on metal-doped activated-carbon catalysts for biodiesel production has increased steadily from 2005 to 2025, illustrated in Fig. 3, with the most pronounced growth occurring after 2015. This trend reflects increasing interest in sustainable heterogeneous catalysis, waste-derived carbon supports, and process optimization for biodiesel production. Recent papers show lower citation counts mainly because of publication recency.

**Fig. 3:**
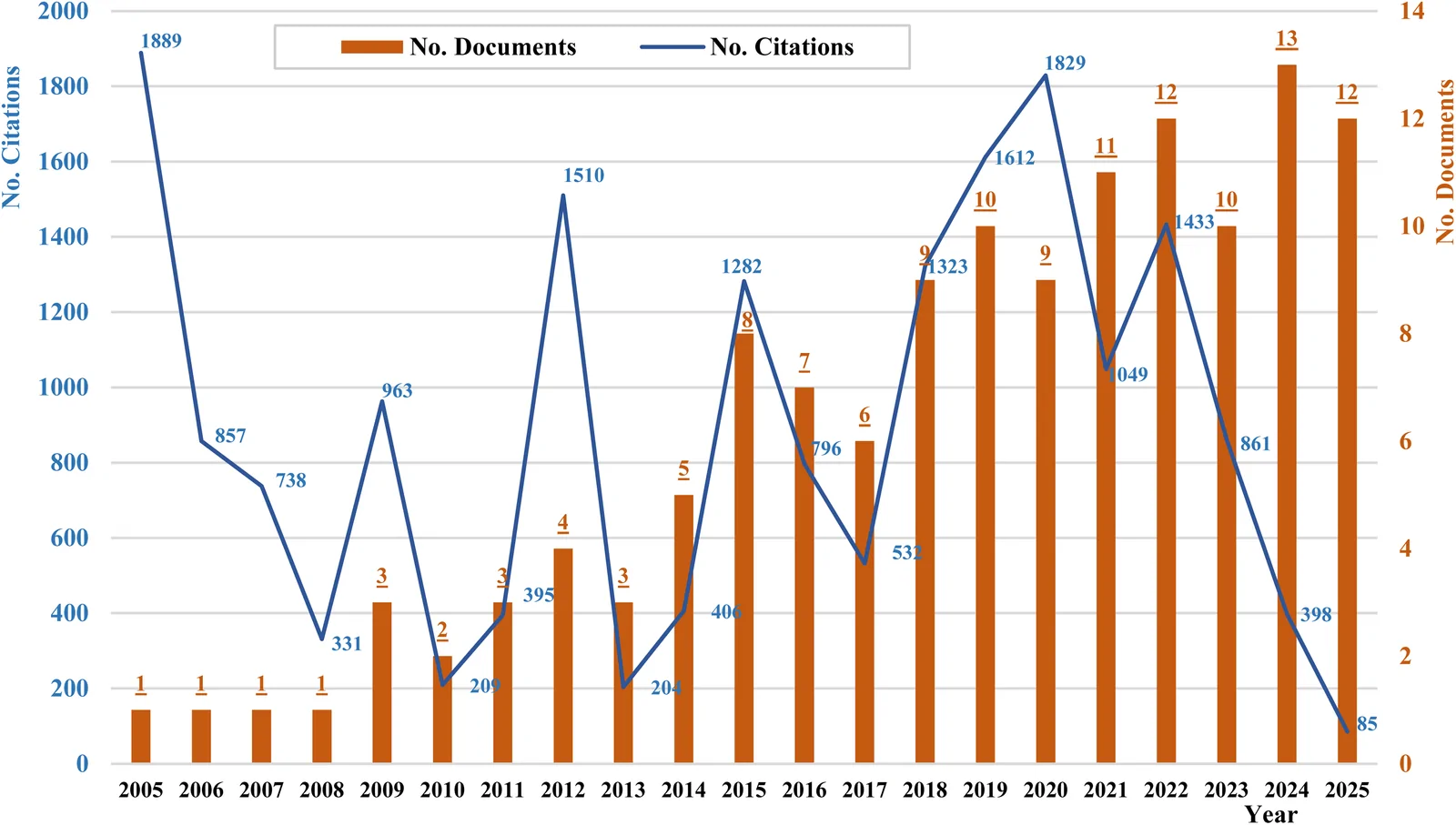
Annual trends in publications and citations on metal-doped activated-carbon catalysts for biodiesel production.

### 3.2 Keywords and countries network analysis

Keyword network analysis provides a powerful approach to identify research trends, key themes, and interconnections in the field of metal-doped AC catalysts for biodiesel production. By analyzing co-occurring terms, researchers can pinpoint emerging topics, highlight influential concepts, and guide future investigations in catalyst design and process optimization. Fig. 4, generated using VOSviewer, illustrates the 47 most frequently co-occurring keywords, with a minimum occurrence threshold of 20. In the network, each node represents a keyword. Node size reflects keyword occurrence or weight, node color denotes VOSviewer cluster membership, and links represent co-occurrence relationships between terms.

**Fig. 4:**
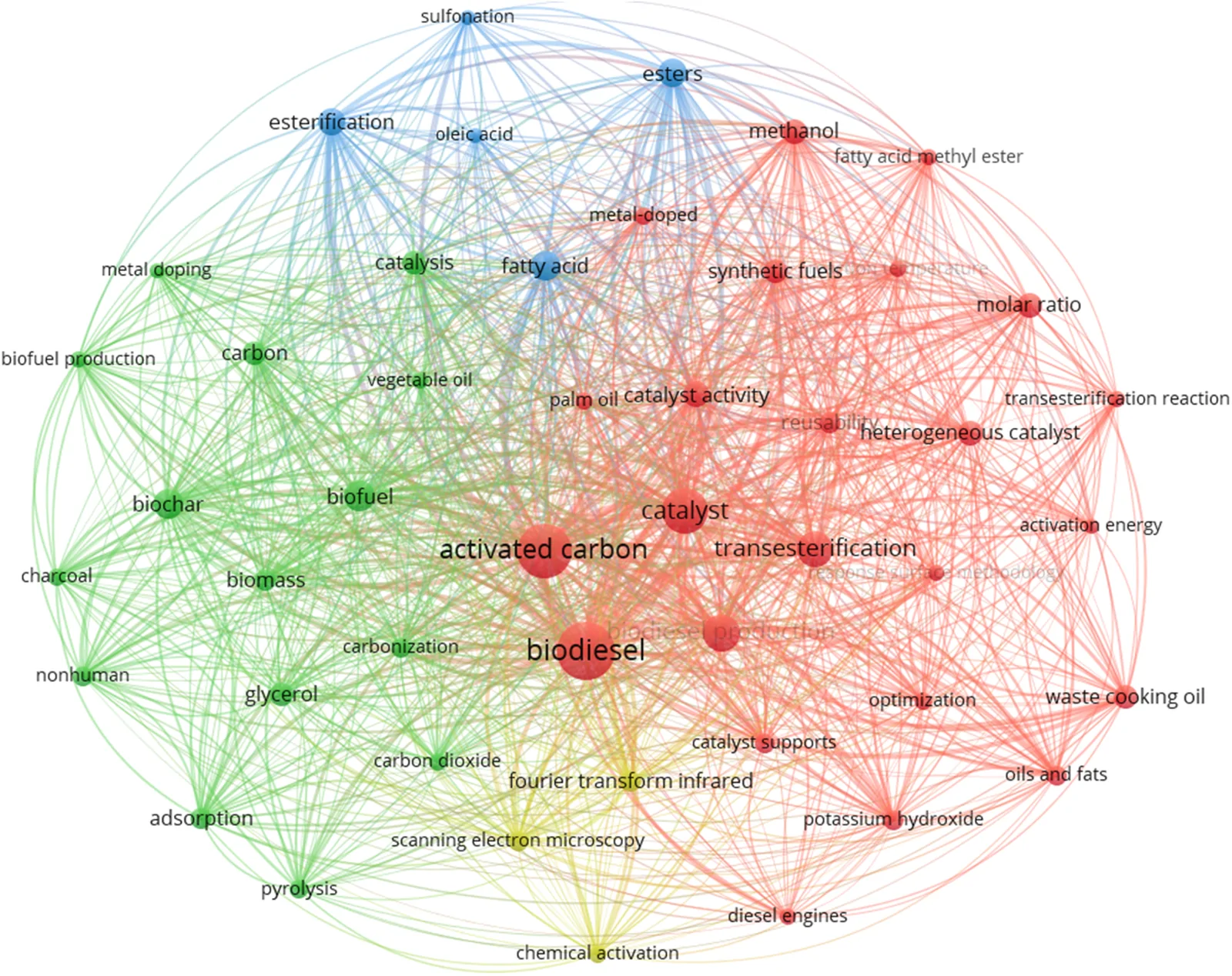
Network of the most frequently used keywords in the reviewed studies on biodiesel catalysts.

The keyword network reveals four main clusters in biodiesel research. The yellow cluster centers on catalyst preparation and characterization, including chemical activation, scanning electron microscopy (SEM), and Fourier transform infrared analysis. The blue cluster relates to feedstock and reaction chemistry, such as esters, fatty acids, oleic acid, sulfonation, and transesterification. The green cluster highlights biofuel production, adsorption, and biomass conversion, covering biochar, carbonization, catalysis, pyrolysis, glycerol, and vegetable oils. The red cluster represents catalyst performance, process optimization, and biodiesel applications, including activated carbon, catalyst activity, metal-doped catalysts, reusability, optimization, and waste cooking oil. Overall, the network captures the field's main research themes and the factors that guide the design and optimization of metal-doped AC catalysts for biodiesel production.

Analyzing the countries contributing to research on metal-doped AC catalysts for biodiesel production helps identify key collaborators and reveal global research patterns. As shown in Fig. 5, the network generated with VOSviewer included countries with at least 10 publications, with 17 countries that met this threshold. Each node represents a country, sized according to its number of published studies. The collaboration network indicates that research on metal-doped AC catalysts is internationally distributed, with particularly strong contributions from Asian countries and growing cross-country collaboration. This pattern reflects the global relevance of biomass valorization, waste-to-fuel strategies, and catalyst development for sustainable biodiesel production.

**Fig. 5:**
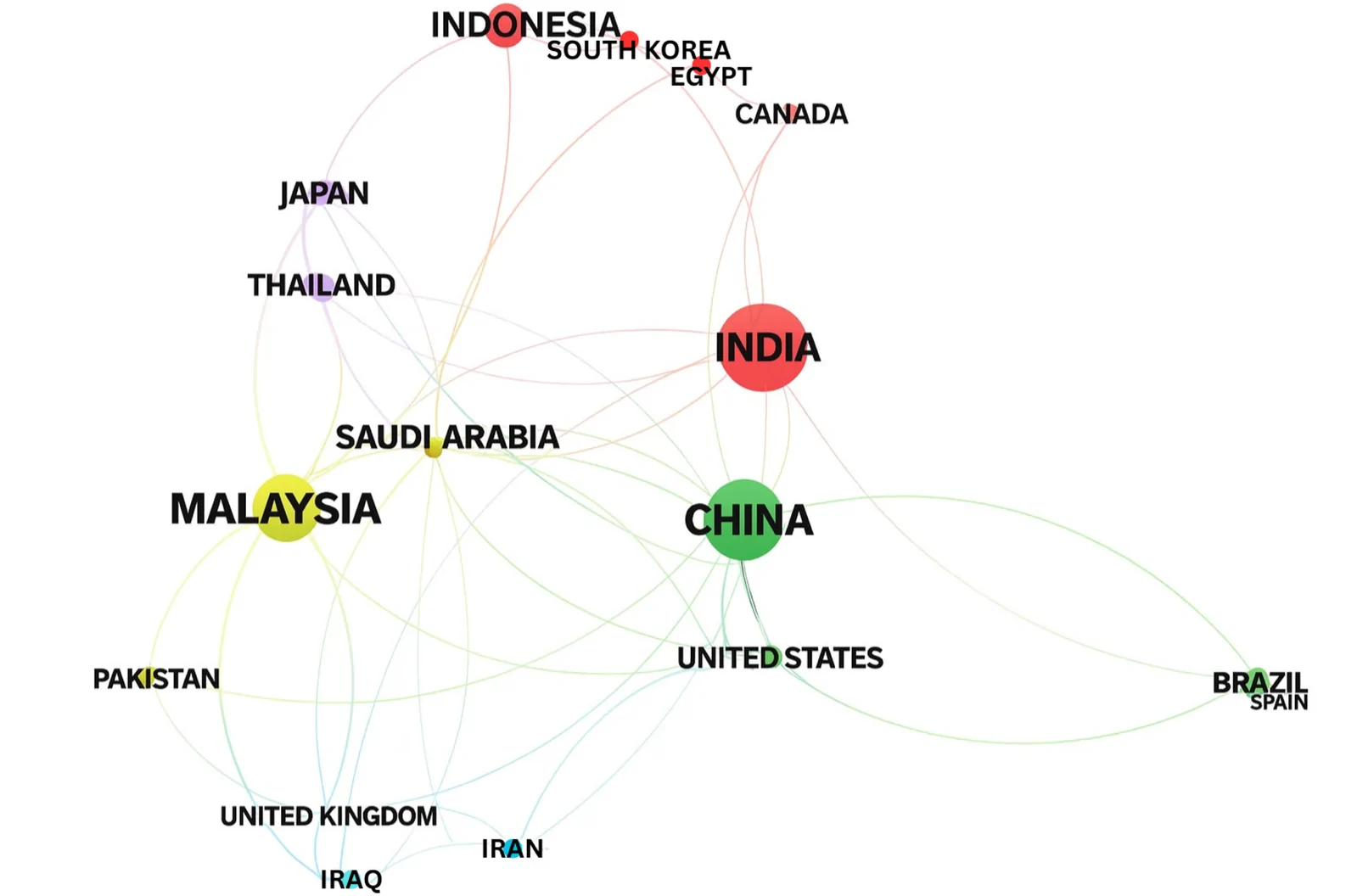
Collaboration network of contributing countries in biodiesel catalyst research.

## 4. Systematic review results

As the second component of the proposed analytical framework, the systematic review was conducted using the final set of selected papers and organized around themes directly aligned with the scope of this study. Specifically, the review covers: (1) biodiesel feedstocks, (2) feedstock pretreatment, (3) biodiesel production processes, (4) catalysts in biodiesel production, (5) factors affecting biodiesel production, (6) physicochemical properties of biodiesel, (7) characteristics of biodiesel produced using metal-doped biochar, and (8) modelling of biodiesel production, including both conventional approaches and machine learning models for metal-doped activated carbon systems. These themes are integrated through the proposed catalyst–machine learning framework, linking catalyst properties, feedstock characteristics, process conditions, and catalytic performance with ML-based prediction and optimization. Collectively, these sections provide a focused synthesis of how catalyst design and data-driven methods can advance sustainable biodiesel production.

### 4.1 Biodiesel feedstocks

Biodiesel can be produced from edible oils, non-edible oils, waste lipids, animal fats, and algal biomass; however, from the perspective of this review, the most important distinction among feedstocks is not their generation classification but rather their free fatty acid content, moisture level, impurity profile, availability, and cost.^27–29^ These properties directly influence pretreatment requirements, catalyst suitability, and the feasibility of one-step *versus* two-step conversion routes.^30,31^ In particular, waste-derived and non-edible feedstocks are attractive because they reduce competition with food resources, but they often require more robust catalytic systems due to higher variability and FFA content. Accordingly, feedstock is considered here primarily as a determinant of metal-doped AC/biochar catalyst selection and process optimization.^15,32,33^ As shown in Fig. S1 in the SI, feedstocks are classified into four generations based on their origin, sustainability, and implications for food supply chains.

### 4.2 Pretreatment of feedstocks

Pretreatment is mainly required for feedstocks with elevated free fatty acid and moisture contents, especially waste cooking oils and low-quality non-edible oils. When FFA levels are high, direct alkaline transesterification becomes inefficient because of soap formation, making acid esterification or bifunctional catalytic approaches more suitable. In the context of this review, pretreatment is important because it determines whether metal-doped AC catalysts should be designed as basic, acidic, or bifunctional systems for efficient biodiesel production.^34–36^

### 4.3 Biodiesel production process

Biodiesel can be produced from renewable feedstocks such as vegetable oils, animal fats, and waste cooking oils using several routes, including micro-emulsification, dilution, catalytic and non-catalytic transesterification, and intensified methods such as microwave- and ultrasonication-assisted processes.^37–39^ Among these, transesterification is the dominant route because of its cost-effectiveness and scalability.^38,40^ In this process, triglycerides react with alcohol, usually methanol, to produce FAMEs and glycerol. Homogeneous catalysts are inexpensive and highly active but require extensive purification, whereas heterogeneous catalysts offer easier separation and cleaner products, though often at a higher cost.^4^

To overcome mass-transfer limitations in heterogeneous systems, microwave irradiation and ultrasonication have been applied to improve heating, mixing, and reaction rates.^37,41^ Supercritical methanol transesterification is another alternative that achieves high conversion rapidly without a catalyst, but it requires high temperature, high pressure, and significant energy input.^42,43^ In catalytic transesterification, maintaining anhydrous conditions is essential to avoid saponification and preserve product quality.^34^ A comparative summary of these production routes is provided in Table 3, while Fig. 6 schematically presents the principal biodiesel-production sequence, including feedstock pretreatment when required, alcohol and catalyst addition, esterification/transesterification, phase separation, biodiesel purification, glycerol-rich by-product recovery, and alcohol recovery/recycle. Overall, process selection depends on feedstock properties, catalyst type, operating conditions, and downstream separation requirements.

**Table 3:** Summary of biodiesel production techniques: advantages and limitations

Technique	Advantages	Limitations
General transesterification	Produces biodiesel with properties similar to petroleum diesel	Requires low FFA, low moisture, and clean feedstocks; impurities must be removed
Homogeneous catalysis	Fast conversion, simple operation, scalable, and cost-effective	The catalyst is not reusable; soap formation may occur; glycerol by-product quality is low
Heterogeneous catalysis	High biodiesel yield; easier purification; catalyst can be recovered and reused	Higher cost; slower reaction rates
Enzyme catalysis	Works well with high FFA/moisture; no soap formation; energy-efficient	Enzymes are expensive; methanol can inhibit activity, and reaction times are longer
Supercritical/non-catalytic process	No catalyst required; simple purification; fast reactions	Requires very high temperature and pressure; high methanol use and energy demand

**Fig. 6:**
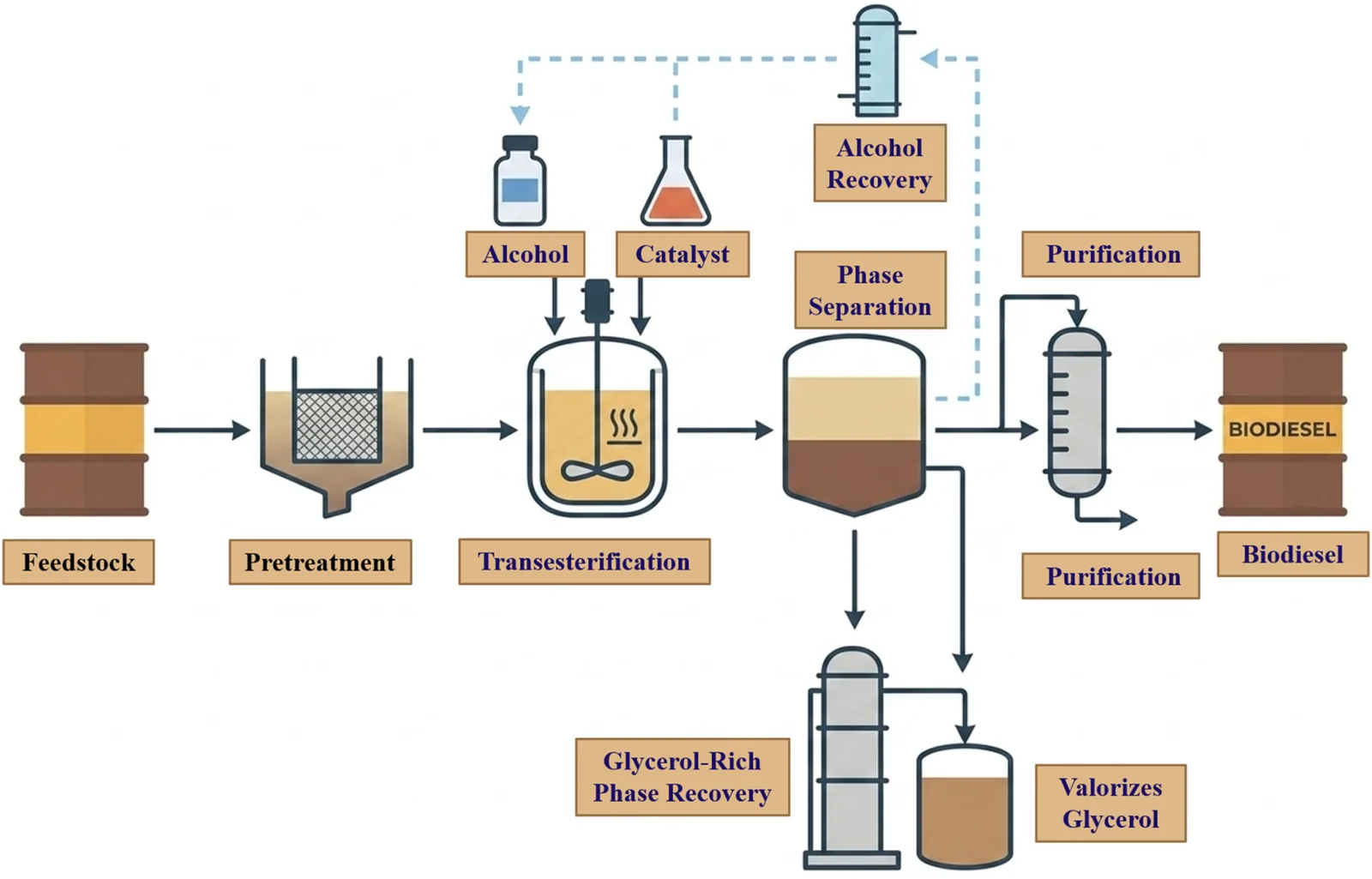
Schematic of the biodiesel production process and downstream product recovery.

### 4.4 Catalysts in biodiesel production

#### 4.4.1 Homogeneous *vs.* heterogeneous catalysts

Catalysts enhance biodiesel transesterification by accelerating reaction rates without being consumed, although their efficiency may decline through repeated use.^5^ Based on their phase relative to the reaction mixture, catalysts are classified as homogeneous or heterogeneous.^44,45^

Homogeneous catalysts (acidic or basic) operate in the same liquid phase as the oil and alcohol and are valued for their high reaction rates, good selectivity, and adjustable activity.^8,46^ Acid catalysts such as H_2_SO_4_ and RSO_3_H are well suited to high-FFA feedstocks due to their resistance to soap formation, though they suffer from slow kinetics and corrosion issues during downstream purification.^6,8,29,47^ Alkaline catalysts (NaOH, KOH, CH_3_ONa, CH_3_OK) are favoured industrially because they offer fast kinetics, low cost, and operate under mild conditions.^48^ However, for feedstocks with FFA contents above 1 wt%, they generate soap and reduce yield; therefore, a two-step esterification–transesterification sequence is usually adopted.^49,50^

Heterogeneous catalysts remain in the solid phase and offer advantages such as ease of separation, reusability, lower environmental impact, and compatibility with a wide range of feedstocks.^51,52^ Acidic heterogeneous catalysts (Nafion, zeolites, and sulphated zirconia) tolerate FFAs but react more slowly,^16,52^ whereas basic heterogeneous catalysts (CaO, MgO, SrO, and hydrotalcites) provide high yields, fast kinetics, and minimal soap formation, though they may require higher catalyst loadings or regeneration.^17,53^ Moreover, waste-derived catalysts such as those derived from eggshells, banana peels, and sugarcane bagasse offer low-cost, sustainable alternatives due to their naturally high metal oxide content.^18,54,55^

#### 4.4.2 Activated carbon as catalyst support

From this point onward, the review concentrates on carbon-supported and metal-doped catalyst systems, since these materials represent the central scientific focus of the manuscript and the clearest opportunity for performance enhancement in sustainable biodiesel production. Activated carbon (AC) can function not only as a catalyst precursor but also as a support for dispersed acidic, basic, and metal-containing active phases. Its tunable surface chemistry, pore architecture, and high surface area make it suitable for anchoring catalytically active species, while the use of waste-derived precursors provides additional environmental and economic advantages.^56^Table 4 summarizes representative waste-derived AC systems, including shea nut shells, spent coffee grounds, beechwood biomass, and date-seed waste converted into KOH-activated carbon catalysts. Although the reported surface areas range from 72 m^2^ g^−1^ for beechwood biomass to 669.3 m^2^ g^−1^ for spent coffee grounds, the reported biodiesel yields remain high (91.6–97.6%). However, these values should not be interpreted as a direct ranking of catalyst quality because the studies differ in feedstock, catalyst preparation, alcohol, and reaction conditions.^8,57^

**Table 4:** Activated carbon-derived heterogeneous catalysts from wastes for biodiesel production

Waste-derived precursor	Activating agent	Surface area (*S*_BET_, m^2^ g^−1^)	Feedstock oil	Alcohol employed	Biodiesel yield (%)	Ref.
Shea nut shells	KOH	—	*Jatropha curcas* oil	Ethanol	97.6	57
Spent coffee grounds	KOH	669.3	Waste cooking oil	Methanol	91.6	65
Beechwood biomass	KOH	72	Corn oil	Methanol	92.0	8
Date seed waste	KOH	260	Waste oil	Methanol	93.0	66

The evidence base extends beyond the KOH-activated systems summarized in Table 4. KOH supported on palm-shell activated carbon has been investigated for palm-oil transesterification,^58^ while Na/Ca-supported AC systems have been prepared through controlled impregnation and thermal treatment.^59^ CaO supported on waste-shell-derived AC provides another example of a waste-derived carbon support combined with a basic active phase.^60^ Other studies illustrate the broader chemical flexibility of AC-supported systems, including Fe-doped carbon catalysts for biodiesel production,^61^ bimetallic Fe–Zn/AC, Cu–Zn/AC, and Cu–Fe/AC systems,^62^ lithium supported on activated carbon derived from scrap tires,^63^ and carbon-supported MgO catalysts.^64^ Together, these studies demonstrate that AC can accommodate different active phases and catalytic functions rather than serving solely as an inert high-surface-area carrier.

The catalytic role of the carbon support depends on the combined effects of precursor origin, activation route, surface functionality, pore structure, active-phase identity and loading, and metal–support interactions. Accordingly, meaningful comparison of AC-supported catalysts should consider support characteristics together with active-phase composition, reaction conditions, catalytic performance, and reuse or leaching behavior. This broader descriptor set is also relevant to the catalyst-aware machine-learning framework developed later in this review.

Many review articles (*e.g.*, ref. 67 and 68) have addressed carbon-based catalysts for biodiesel production; however, their coverage is largely generic and often lacks depth regarding catalyst design from agricultural residues. Most studies focus on broad carbon materials (*e.g.*, carbon nanotubes, graphene oxide, biochar, and activated carbon) without critically distinguishing waste-derived precursors or their structure–property relationships. For instance, ref. 54 includes various carbon sources such as carbohydrates and petroleum-derived materials but gives limited attention to agricultural waste as a sustainable feedstock. In addition, functionalization strategies (acid, base, or enzyme modification) and their mechanistic impact on catalytic performance remain insufficiently analyzed. Therefore, this review critically addresses this gap by focusing on the conversion of agricultural waste into functionalized biochar and activated carbon for biodiesel production.

#### 4.4.3 Metal doping of activated carbon

Metal-functionalized biochar (M-BCH) catalysts for biodiesel production are typically prepared using two main approaches, as shown in Fig. 7. In the one-step (*in situ*) method, a metal precursor is mixed with the biomass before pyrolysis, enabling simultaneous biochar activation and metal nanoparticle formation, which enhances simplicity and scalability. The two-step (post-activation) method produces the biochar first and subsequently impregnates it with metal salts, offering greater control over nanoparticle characteristics but with increased procedural complexity.^67^

**Fig. 7:**
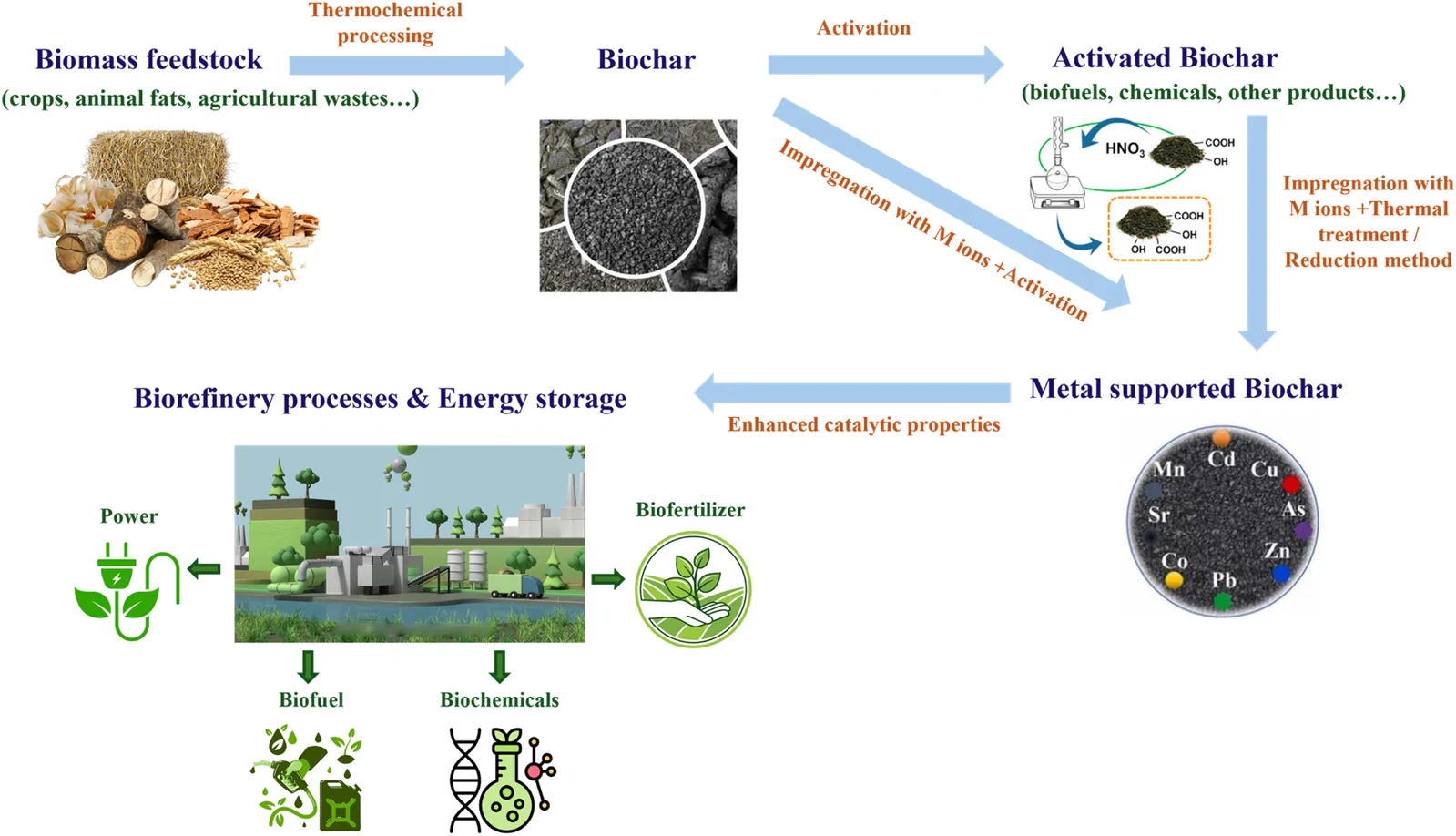
Overview of metal-supported biochar synthesis routes.

Metal functionalization does not confer a single catalytic function on biochar or activated carbon. K-containing phases (*e.g.*, KOH/K_2_CO_3_) and CaO mainly provide basic functionality for transesterification, whereas Ni- and Fe-containing phases can exhibit different catalytic or functional roles depending on their chemical form and the process being studied. In the Na/Ca-supported AC system,^69^ the manuscript identifies Na_2_CO_3_ and CaO as catalytically relevant phases. The K/Ni rice-husk catalyst^70^ combines K-containing basic functionality with magnetic recoverability (see Fig. 8), while the Fe-containing AC systems discussed in ref. 71 and 72 are associated with high-temperature catalytic cracking rather than being treated as generic solid-base transesterification catalysts. Accordingly, catalyst performance should be attributed to the identified active phase and its specific function, rather than to metal functionalization as a uniform effect. Despite the high catalytic performance reported for several of these systems, their practical deployment remains constrained by challenges related to catalyst stability, reusability, and metal leaching during reaction.

**Fig. 8:**
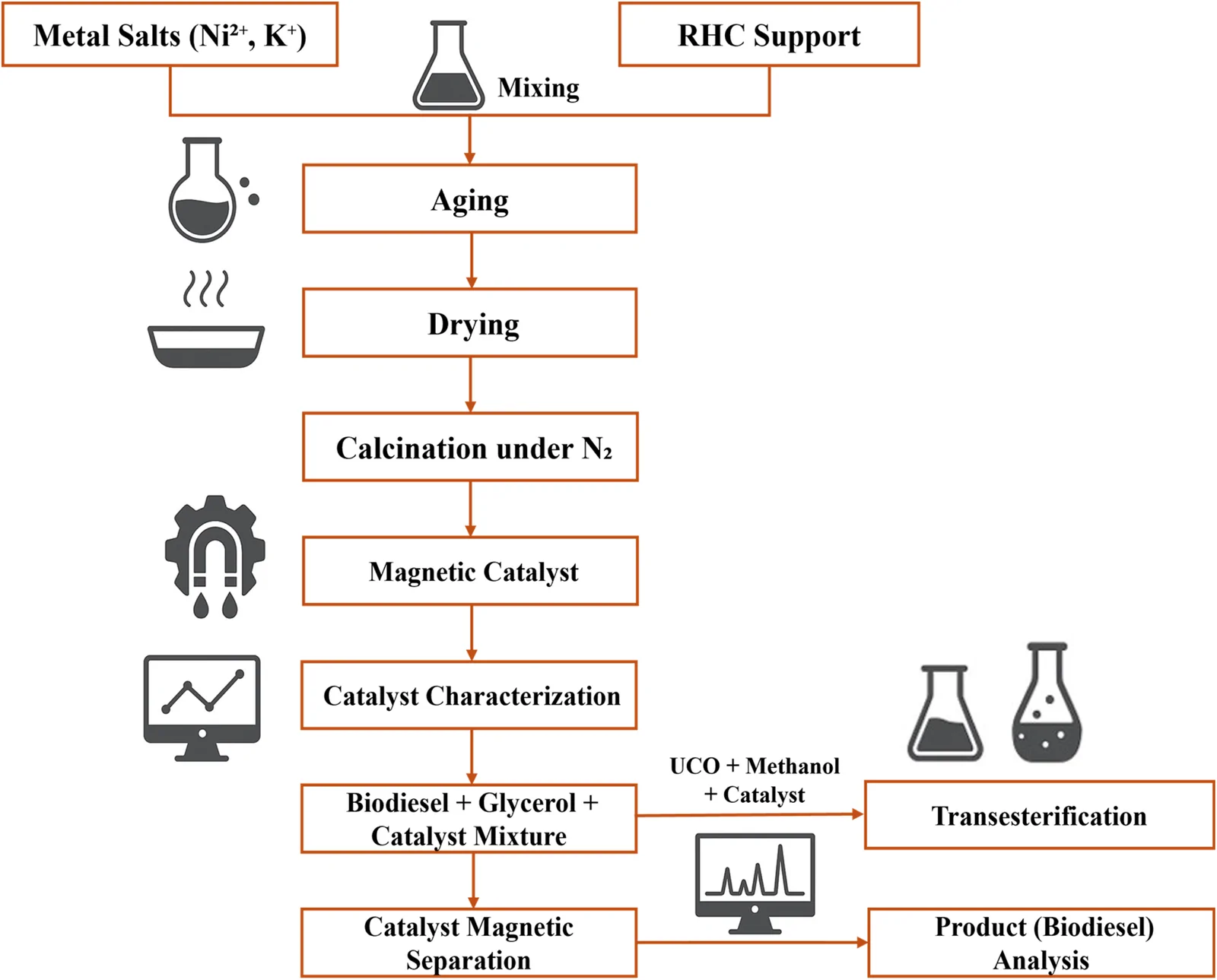
A rice husk–derived K–Ni functional biochar catalyst for WCO transesterification.

Representative waste-derived biochar catalysts and their transesterification performance are summarized in Table 5. Several K- and Ca-containing systems achieve high FAME yields under optimized reaction conditions, while the Ni–KOH rice-husk catalyst demonstrates the potential for combining catalytic functionality with magnetic recoverability.

**Table 5:** Summary of biochar-based catalysts derived from various feedstocks

Biochar feedstock	Catalyst M-support	Substrate	Methanol-to-oil ratio	Temperature (°C)	Time (h)	FAME yield (%)	Ref.
Peat	KOH	Palm oil	8 : 1	65	1.5	98.6	70
Pomelo peel	KOH	Palm oil	8 : 1	65	2.5	98.0	73
Flamboyant pods	KOH	*Hevea brasiliensis* oil	15 : 1	60	1	89.3	74
Peat	KOH–CaO	Palm oil	8 : 1	65	2.5	93.4	68
Avocado seeds	CaO	Sunflower oil	15.6 : 1	99.5	5	96.0	75
Bamboo powders	Ni	Soybean oil	9 : 1	65	2	98.1	76
Rice husk	Ni–KOH	Cooking oil	12 : 1	65	2	98.2	70

The metal-doped activated-carbon studies involving catalytic cracking are considered separately from the transesterification systems because these processes involve different reaction pathways, operating temperatures, products, and performance indicators. In ref. 71, Mn/Fe-modified AC prepared from ZnCl_2_-activated peach seed shells was investigated for catalytic cracking at 250–260 °C and was associated with improved fuel quality and an increase in brake thermal efficiency under BF10 operation. Similarly, ref. 72 reported Mn/Fe-doped KOH-activated cellulosic AC for catalytic cracking at 240–250 °C, with improved biofuel characteristics and a 14.5% reduction in brake-specific fuel consumption. These two studies are therefore summarized separately in Table 6, with emphasis on catalytic cracking and engine-related performance indicators. Both studies, however, provided limited information on catalyst recyclability and long-term stability under the relatively high operating temperatures employed.

**Table 6:** Comparative evaluation of metal-doped activated carbon catalysts from agricultural wastes for biofuel production

Feedstock	Activation method	Doped metal	Process type	Best catalyst	Yield	Engine performance	Reusability	Ref.
Peach seed crusts	ZnCl_2_	Mn/Fe	Catalytic cracking (250–260 °C)	Fe-PAC	—	BF10 improved BTE by 11%	—	71
Cellulosic waste	KOH	Mn/Fe	Catalytic cracking (240–250 °C)	Fe/ACCW	—	BF10 reduced BSFC by 14.5%	—	72

In contrast, the CaO/activated-carbon system reported in ref. 77 was evaluated for transesterification at 65 °C and is therefore considered alongside other waste-derived transesterification catalysts. The catalyst achieved 96% FAME yield and maintained stable performance over 10 cycles, with minimal Ca^2+^ leaching. These results support the role of CaO as a relevant basic phase for transesterification. However, engine testing was not evaluated, despite the reported fuel properties meeting ASTM D6751 and EN 14214 standards. The CaO/AC system and other waste-derived transesterification catalysts are summarized in Table 7 using process-specific indicators such as FAME yield, catalyst reusability, metal leaching, and operating conditions.

**Table 7:** Comparative evaluation of waste-derived catalysts used for transesterification

Catalyst	Strengths	Weaknesses	Performance	Operational notes	Ref.
CaO/AC from peach shells	High FAME yield; mild reaction temperature; reuse data available	<15% yield decrease over 10 cycles; engine testing not evaluated	96% FAME; minimal Ca^2+^ leaching; 10 cycles	65 °C; 20% CaO/AC	77
WBWP	Low-cost CaO; simple calcination; good stability; environmentally friendly	Sintering risk at high *T*; moderate surface area	92.7% yield; *E*_a_ = 25.95 kJ mol^−1^; stable over 6 cycles	Works at mild conditions (64 °C, 1.3% loading)	78
EFB	Very high reported surface area; bifunctional; suitable for high-FFA oils	Catalyst deactivation after several uses; complex synthesis	97.1% yield; >80% retained after 5 cycles	Requires higher catalyst loading (5%) & MeOH ratio	59

Two additional studies further demonstrate the application of waste-derived catalysts in transesterification. The white brick waste powder (WBWP) catalyst,^78^ obtained by calcining construction waste at 700 °C, produced CaO as the active phase, as confirmed by TGA, XRD, FTIR, BET, and SEM analyses. It achieved a 92.7% yield under relatively mild conditions (1.3% catalyst loading, 64 °C, 5.5 : 1 methanol-to-oil ratio, and 139 min) and exhibited stable performance over six cycles, with an activation energy, *E*_a_ = 25.95 kJ mol^−1^. Similarly, the EFB-derived catalyst,^59^ synthesized by hydrothermal carbonization and modified with K_2_CO_3_ and Cu(NO_3_)_2_, exhibited bifunctional catalytic behavior and a very high reported surface area of 4056.17 m^2^ g^−1^. It achieved a 97.1% yield at 5% catalyst loading, 70 °C, and a 12 : 1 methanol-to-oil ratio, while retaining more than 80% of its activity after five cycles. BET, TPD, FESEM, and FTIR analyses confirmed its porous structure and suitability for high-FFA feedstocks.

Moreover, some studies highlight AC-supported catalysts for biodiesel production, emphasizing Ca- and Na-based systems and tunable carbon materials, as summarized in Table 8. In ref. 69, Na- and Ca-supported AC catalysts were prepared by wet impregnation followed by thermal treatment over 500–800 °C. The reported 700–800 °C range refers to catalyst heat-treatment temperature, not to the biodiesel reaction temperature; catalysts treated in this range were associated with high biodiesel yields and minimal metal leaching. ^23^Na NMR confirmed Na_2_CO_3_ and CaO phases. The review^19^ stressed that porosity, acidity/basicity, and surface hydrophilicity can be engineered in biochar, AC, mesoporous carbon, and carbon nanotubes to enhance esterification and transesterification, especially for high-FFA oils. In the study,^60^ a CaO–AC catalyst from mollusk shells produced >90% biodiesel at 60 °C in 6 h, maintaining activity over five cycles.

**Table 8:** Activated-carbon-based catalyst systems experimentally evaluated for biodiesel production

Catalyst	Strengths	Limitations	Key innovation	Ref.
(Na, Ca)/AC	Strong basicity, low metal leaching, thermally stable, good reusability	Requires high calcination temperature; exact yield not reported	Use of ^23^Na NMR to study structural changes: a unique analytical advantage	69
AC–CaO from waste shells	Fully waste-derived, low cost, environmentally friendly, strong basicity	Slight deactivation; high calcination temperature (900 °C); CO_2_ adsorption sensitivity	“Green” circular-economy catalyst with full waste valorisation	60

Overall, metal doping significantly improves AC/biochar catalytic activity by increasing active-site density and enabling functionalities such as bifunctionality or magnetic recovery. However, these benefits are often offset by issues such as pore blockage, metal leaching, and limited durability data. This performance–stability trade-off remains a key challenge and an important target for future ML-driven optimization.

#### 4.4.4 Biochar *versus* metal-doped biochar

Biochar is a carbon-dense material produced through pyrolysis of biomass under oxygen-limited conditions and has gained significant attention as a sustainable catalyst and catalyst support in green chemistry applications. It is often derived from agricultural residues such as sugarcane bagasse, rice husk, or wood chips. Biochar promotes a circular economy by converting low-value biomass into functional materials.^79^ Pristine biochar exhibits moderate surface area, tunable porosity, thermal and mechanical stability, and surface oxygenated groups (*e.g.*, carboxyl, hydroxyl, carbonyl) that impart mild acidity and enable chemical interactions.^20^ However, its catalytic performance in reactions like triglyceride transesterification is limited, as inherent ash content and weak acid–base sites are insufficient for high conversion under mild conditions.^20^ While useful as an adsorbent or support, biochar requires further functionalization to serve as an efficient standalone catalyst in biodiesel production.

Incorporating metals into biochar transforms it from a passive support into an active catalytic composite. Metal-doped biochars are prepared by impregnating biomass or the resulting biochar with metal precursors, such as alkaline earth metals (Ca, Mg), transition metals (Fe, Ni, Cu), or rare earth metals, followed by thermal treatment.^10,80^ The biochar scaffold provides a high surface area and stabilizes dispersed metal sites, enhancing catalyst activity. Metal doping introduces strong, well-defined active sites; for example, CaO-doped biochar functions as a robust solid base for transesterification, while sulfonated or sulfated metal-doped biochars act as solid acids, enabling simultaneous esterification and transesterification of high-FFA feedstocks.^10,20^ This design flexibility allows the creation of bifunctional catalysts, providing a potential advantage over pristine biochar.

Metal-doped biochar outperforms pristine biochar due to several factors:

• Catalytic activity and yield: metal incorporation enhances the density and strength of active sites, enabling biodiesel yields often exceeding 90–95% under milder conditions and shorter reaction times, whereas pristine biochar typically gives lower conversions.^79^

• Textural property enhancement: metal doping can modify biochar structure, as some metals act as porogens during pyrolysis, increasing porosity and surface area, thereby improving reactant accessibility to active sites.^80^

• Novel functionalities: magnetic metals such as Fe_3_O_4_ impart magnetic properties, allowing easy catalyst recovery with an external magnet and improving operational reusability.^10,20^

• Stability and reusability: the biochar matrix stabilizes metal nanoparticles, preventing aggregation or leaching during repeated cycles, resulting in sustained catalytic performance.^67^

Despite their advantages, metal-doped biochars present challenges not associated with pristine biochar. The main concern is metal leaching, particularly for basic catalysts like CaO, which can react with FFAs to form soap, potentially deactivating the catalyst and contaminating the biodiesel product, thereby requiring additional purification.^79^ Synthesis is also more complex and costly, involving metal impregnation, additional calcination, and the consumption of metal salts, unlike the simpler one-step pyrolysis of pristine biochar. Improper control of the synthesis conditions can lead to pore blockage, reducing surface area and limiting reactant access.^80^

In summary, pristine biochar offers simplicity, low cost, and sustainability, suitable for applications with modest catalytic demands. Metal-doped biochar, by contrast, is engineered for high activity, yield, and advanced features such as magnetism and bifunctionality, but incurs higher synthesis complexity and potential leaching. Selecting between the two depends on balancing catalytic performance, economic considerations, and sustainability, particularly when processing high-FFA feedstocks for biodiesel.

#### 4.4.5 Catalyst physicochemical properties

In solid acid-catalyzed FFA esterification, catalyst performance is governed by the density and accessibility of acid sites, textural properties, and mass-transfer characteristics. High Brønsted acidity is essential for protonating free fatty acids and activating the carbonyl group, thereby accelerating esterification kinetics. Meanwhile, mesoporous structures with large pore diameters facilitate the diffusion of bulky FFA and triglyceride molecules, minimizing internal diffusion limitations and improving reaction rates.

High surface area increases the number of accessible active sites, while adequate pore volume enhances reactant–catalyst contact. In addition, hydrophobic carbon frameworks promote preferential adsorption of organic reactants over water, reducing catalyst deactivation and shifting the equilibrium toward ester formation. Together, these physicochemical properties directly influence biodiesel yield, reaction efficiency, and catalyst stability.^19^

Solid acid catalyst performance in FFA esterification is strongly governed by surface area, porosity, and acid site density. Sulfonated activated carbon (ACPhSO_3_H) offers mesoporosity (with a surface area of 114 m^2^ g^−1^ and a pore size of 10.1 nm) with moderate acid density (0.98 mmol H^+^ g^−1^), while Amberlyst-15 provides a lower surface area (40 m^2^ g^−1^) but higher acid density (4.64 mmol H^+^ g^−1^) and larger pores (32.9 nm). Despite these differences, both catalysts achieve similar FFA conversion (∼95% in 7 h), indicating that well-designed bio-based catalysts can achieve performance comparable to that of commercial resins under optimized conditions.^19,60,81^

The thermal stability of ACPhSO_3_H was verified by TGA, which showed SO_3_H and PhSO_3_H desorption and decomposition between 150–400 °C, confirming successful functionalization. FT-IR detected sulfonic groups, and XRD indicated that the carbon support remained amorphous after modification. Nitrogen sorption revealed major reductions in surface area (∼81%) and pore volume (∼61%) due to micropore blockage by grafted groups. FE-SEM showed a meso-/macroporous structure favorable for the diffusion of molecules <4 nm. The catalyst showed moderate recyclability, with a 32% activity drop after seven cycles due to PhSO_3_H leaching.^81^ The reported regeneration and activity-recovery behavior is discussed separately in Section 4.4.6.

Recent studies evaluated peach-seed-derived activated carbon (PAC), which was ZnCl_2_-activated and doped with Fe or Mn, for the catalytic cracking of waste cooking oil. PAC exhibited a surface area of 429 m^2^ g^−1^, a pore radius of 2.31 Å, and a pore volume of 0.25 cm^3^ g^−1^. Metal doping slightly increased the surface area (Mn-PAC: 456 m^2^ g^−1^; Fe-PAC: 435 m^2^ g^−1^) but reduced pore size and volume, indicating partial pore blockage and metal incorporation.^81,82^ FTIR confirmed the presence of oxygenated functional groups, while XRD showed preserved crystallinity in Mn-PAC but increased amorphous character in Fe-PAC, suggesting the formation of more defect sites. Both doped catalysts showed improved thermal stability. Fe-PAC, with higher microporosity, delivered the best cracking performance, producing higher-quality bio-oil and, in engine tests, BF10 blends with reduced fuel consumption and higher brake thermal efficiency compared with diesel.^71^

To contextualize the preceding discussion, Table 9 summarizes the key physicochemical properties, stability, and catalytic performance of ACPhSO_3_H, Amberlyst-15, and metal-doped activated carbons (PAC, Mn-PAC, Fe-PAC). This comparison illustrates how variations in surface area, pore architecture, acidity, and thermal robustness govern catalytic activity, product quality, and recyclability in biodiesel production.

**Table 9:** Performance and material characteristics of ACPHSO_3_H, Amberlyst-15, and metal-doped PAC catalysts in biodiesel applications

Catalyst system	Surface area (m^2^ g^−1^)	Pore characteristics	Acid density (mmol H^+^ g^−1^)	Thermal stability	Catalytic performance	Reusability/engine results	Ref.
ACPhSO_3_H	114	Mesoporous, 10.1 nm	Total: 0.98; PhSO_3_H: 0.72	Stable up to 400 °C; SO_3_H decomposition observed	∼95% FFA conversion (esterification) comparable to Amberlyst-15	32% loss after 7 cycles; regenerable	19
							81
Amberlyst-15	40	Macroporous, 32.9 nm	Total/PhSO_3_H: 4.64	High stability	High initial esterification rate; ∼95% FFA conversion after 7 h	Commercial benchmark	81
PAC (peach seed AC)	429	Pore radius: 2.31 Å	—	Stable up to 350 °C	Moderate cracking activity	Higher density/viscosity fuels	75
							82
							81
Mn-PAC	456	Pore radius: 2.23 Å	—	Higher stability than PAC	Better than PAC but lower than Fe-PAC	Moderate performance	75
							82
							81
Fe-PAC	435	Pore radius: 2.15 Å; more microporous	—	High stability; more amorphous	Best cracking activity; lower S, viscosity, density	BF10 improves efficiency, lowers BSFC *vs.* diesel	75
							82
							81

#### 4.4.6 Reusability, deactivation, and regeneration of waste-derived carbon catalysts

Reusability and regeneration should be distinguished when evaluating the long-term performance of waste-derived carbon catalysts. Reusability refers to retaining catalytic activity after catalyst recovery and reuse in subsequent reaction cycles, whereas regeneration involves an additional treatment intended to restore catalytic activity following deactivation. Among the systems reviewed, the sulfonated activated-carbon catalyst ACPhSO_3_H showed a 32% activity decrease after seven cycles due to PhSO_3_H leaching; regeneration subsequently restored most of the acid sites,^81^ providing direct evidence of activity recovery rather than simple catalyst reuse.

Other studies primarily report catalyst reusability rather than regeneration. The CaO-doped AC catalyst^77^ achieved 96% FAME yield and was reused for 10 cycles with less than 15% yield loss and minimal Ca^2+^ leaching. The EFB-derived bifunctional catalyst retained^59^ more than 80% activity after five cycles, although gradual deactivation associated with pore blockage was observed. Similarly, the AC–CaO catalyst derived from waste shells^60^ maintained its catalytic activity over five cycles, with slight deactivation reported. These findings indicate that catalyst deactivation can arise from different mechanisms, including active-phase or functional-group leaching, pore blockage, and loss of accessible acidic or basic sites.

Within the waste-derived carbon-catalyst studies reviewed here, systematic regeneration studies remain limited. The available literature does not yet provide standardized comparisons of regeneration procedures, regeneration efficiency, and post-regeneration catalyst performance across different catalyst classes. Future studies should therefore report the regeneration or reactivation procedure, catalytic activity before and after regeneration, activity retention over repeated cycles, metal or functional-group leaching where applicable, and post-regeneration characterization such as BET, FTIR, and XRD. Regeneration strategies should be selected according to the identified deactivation mechanism rather than applied as a universal procedure. Such standardized reporting would facilitate more reliable assessment of catalyst lifetime, regeneration efficiency, and practical applicability.

### 4.5 Factors affecting biodiesel production

Reaction temperature, alcohol-to-oil ratio, catalyst loading, reaction time, and feedstock quality strongly influence biodiesel yield; however, in the present review, these variables are considered mainly in relation to catalyst performance.^83^ Across metal-doped AC/biochar systems, the optimal operating window depends on the interplay of catalyst basicity/acidity, pore structure, metal dispersion, and feedstock composition.^84,85^ Accordingly, process variables are discussed here only to clarify differences in catalytic activity, fuel quality, and reusability, whereas the detailed reported optimum operating conditions and corresponding biodiesel yields are provided in Table S1 in the SI.

### 4.6 Physicochemical characteristics of biodiesel

Biodiesel quality is commonly evaluated using viscosity, density, acid value, cetane number, cold-flow behavior, oxidation stability, and flash point, because these parameters determine compliance with ASTM D6751 and EN 14214.^12,86^ In this review, these properties are not discussed as a general fuel-science topic, but rather as performance indicators used to evaluate the effectiveness of metal-doped AC/biochar catalyst systems.

Accordingly, emphasis is placed on studies that connect catalyst formulation and operating conditions to biodiesel properties that meet applicable fuel standards.^87,88^ For broader reference, the physicochemical properties of biodiesel produced from different vegetable oil feedstocks using homogeneous alkali and heterogeneous acid catalysts are summarized in Table S2 in the SI.

### 4.7 Characteristics of biodiesel produced using metal-doped/supported activated-carbon catalysts

#### 4.7.1 Fuel quality and standards compliance

AC serves as a cost-effective, high-surface-area catalyst support with tunable acidity/basicity. Metal doping (*e.g.*, K, Ca, Fe, Zn, Cu) facilitates heterogeneous transesterification of diverse feedstocks under mild conditions, often yielding FAME that meets EN 14214 and ASTM D6751 standards for kinematic viscosity, density, acid value, and cold-flow properties when process conditions and post-treatment are optimized.^89^ Key trends include: (i) K-based basic sites (*e.g.*, KOH/AC) provide high activity and, with proper methanol-to-oil ratios and catalyst loading, produce FAME with viscosity ∼4–5 mm^2^ s^−1^ and density ∼0.88–0.89 g cm^−3^ within standard limits; (ii) incomplete washing or esterification can leave acid values above specification, requiring polishing or blending.^89^

Table 10 summarizes representative studies on metal-doped AC catalysts, comparing catalytic performance, feedstock, and operating conditions alongside the resulting biodiesel properties. This consolidated overview illustrates how catalyst formulation and process parameters influence key fuel characteristics, providing a reference for evaluating the suitability of AC–metal systems in sustainable biodiesel production.

**Table 10:** Comparative characteristics of biodiesel produced using metal-doped activated carbon catalysts

Feedstock	AC-metal catalyst (loading)	Key operating window	FAME yield	Reported biodiesel properties	Standards check/reference
Rapeseed, sunflower, corn, olive oils	KOH on AC (0.5–1.5 wt%); also, lipase/AC	60 °C; MeOH: oil = 6 : 1; 1–4 h	KOH/AC: ∼91–93%; lipase/AC up to 99.9%	Density 885–886 kg m^−3^ viscosity 4.7 mm^2^ s^−1^	Meets EN 14214 under optimized runs.^89^
				CFPP −13 to 4 °C	
Palm oil	KOH/AC (palm-shell AC, BET ≈ 1015 m^2^ g^−1^)	∼70 °C; ∼1 h; RSM-optimized	Up to ∼94%	Properties evaluated; emphasis on efficient heterogeneous base catalysis	Reported as effective; properties discussed *vs.* standards.^58^
Waste frying oil	KOH/AC (packed-bed, *Jatropha*-shell AC)	60 °C; MeOH: oil = 25 : 1; 2 h	86.3% (optimum)	Fuel properties determined for the product from WFO	Authors assess compliance; catalyst reusable × 4
Used cooking oil	KOH/AC (3–5 wt%)	50–65 °C; catalyst 3–5 wt%	Best: 87.51%	Density 0.772–0.859 g mL^−1^ & viscosity 4.55–5.37 cSt & acid value 1.12–2.24 mg_KOH_ g^−1^	Viscosity/density generally within ASTM; acid value above limit → needs polishing/blending.^90^
Rubber seed oil	Fe-doped AC (ferromagnetic Fe/C)	Taguchi-optimized; mild *T*	High conversion (study-dependent)	Properties measured: density, viscosity, acid & iodine values, cetane number	Reported within/near standards after optimization; details in articles.^61^
Sunflower oil	Bimetal/AC: Fe–Zn/AC, Cu–Zn/AC, Cu–Fe/AC	65 °C; ∼3 h; alcohol: oil ≈ 19 : 1; 5 wt% cat	74.7% (Fe–Zn), 89.0% (Cu–Zn), 68.2% (Cu–Fe)	Fuel properties not listed; catalytic comparison	Indicates a strong effect of metal pairing on yield.^62^

ASTM D6751 (B100) and EN 14214 specify kinematic viscosity ranges (approximately 1.9–6.0 and 3.5–5.0 mm^2^ s^−1^ at 40 °C, respectively), as well as density requirements under EN 14214 and a maximum acid number (typically ≤0.5 mg_KOH_ g^−1^ for finished FAME). Several AC-metal systems meet viscosity/density targets directly; acid value non-compliance is usually addressed *via* improved water/methanol removal, resin polishing, or blending.^61^

#### 4.7.2 Catalyst–process relationships and biochar catalyst performance

(i) Basic site strength & dispersion (K on AC): strong, accessible basic sites promote complete conversion of triglycerides and FFAs to FAME, reducing residual mono-/di-glycerides that elevate viscosity and density. High surface-area AC (often >800 m^2^ g^−1^) improves K dispersion and stability.^89^

(ii) Textural properties of AC: micropore/mesopore balance affects mass transfer; studies using palm-/coconut-shell ACs report high BET and good reusability with stable fuel properties.^89^

(iii) Metal identity/pairing (Fe–Zn, Cu–Zn on AC): bimetallic alters basicity/redox characteristics, impacting conversion kinetics and hence final fuel property compliance; yields vary widely with pairing, warranting property testing for each formulation.^62^

(iv) Work-up & polishing: even when conversion is high, insufficient washing/drying can leave acid number above limits; simple polishing brings AV into spec without altering core viscosity/density achieved by the catalyst route.^79^

Using KOH/AC and Fe-doped AC catalysts, FAME was produced within the viscosity and density specifications of EN 14214/ASTM D6751 under mild conditions (60 °C, 1–4 h) with moderate methanol excess (6 : 1 to 25 : 1), consistent with literature reports on AC-metal catalysts. In ref. 89, KOH/AC achieved 91–93% FAME yield, with viscosity ≈4.7 mm^2^ s^−1^ and density ≈885–886 kg m^−3^, while lipase/AC reached a 99.9% yield with similar fuel properties and improved CFPP (−13 °C).

Biomass-derived AC catalysts (palm, coconut, and coal) also produced compliant viscosity/density, though acid values exceeded the specified limits without proper washing, as seen for UCO-derived FAME (AV 1.12–2.24 mg_KOH_ g^−1^; viscosity 4.55–5.37 cSt). These findings highlight that (i) strong basic sites (*e.g.*, K on AC) and (ii) adequate post-treatment are important factors influencing final fuel quality.^89^

Table 11 illustrates reported associations among feedstock characteristics, catalyst properties, and biodiesel performance across different experimental studies. Although many studies reported high biodiesel yields (>90%), the heterogeneous experimental conditions do not establish controlled superiority or robustness of any particular catalyst system. Some multifunctional catalysts containing alkaline or alkaline-earth metals (*e.g.*, CaO, K_2_CO_3_, and Li-based systems) were reported to perform effectively with low-quality waste oils, but their apparent tolerance remains study-specific and dependent on the corresponding feedstock and operating conditions.

**Table 11:** Summary of feedstocks, catalyst characteristics, reaction conditions, and biodiesel properties reported in recent literature

Feature	Feedstocks						
	NEO blend	Castor oil	Waste edible/cooking oil	Used cooking oil	Waste edible oil	Palm oil	Used cooking oil
Study ref.	63	64	91	92	93	68	70
Acid value of feedstock (KOH g^−1^)	0.71	0.1	—	1.26	—	—	0.89
Feedstock_FFA (%)	—	—	0.7	2.53	2.43	—	0.45
Water (%)	—	—	—	0.164	0.11	—	—
Catalyst_name	40Li/WTAC (40% LiOH)	MgO-UREA-800	Biochar/CaO/K_2_CO_3_ catalyst	CFA/ZnO-400	MnFe_2_O_4_@graphene oxide (GO)	30Ca/APB-700	(RHC/K_2_O–20%/Ni–5%)
Metal type	LiOH	MgO	CaO/K_2_CO_3_	ZnO	MnFe_2_O_4_	CaO	K_2_CO_3_ + Ni
Biochar source	Waste tires	Carbon-based	*Sargassum oligocystum*	Coal fly ash	Graphene oxide	Peat	Rice husk char
Metal load in biochar (%)	40	25	50	18.3	—	30	20
Surface_area (m^2^ g^−1^)	306.1	96.1752	3.74	58.76	51.22	70.3	32.4
Pore volume (cm^3^ g^−1^)	—	0.042352	—	0.231	—	0.08524	—
Pore_size (nm)	—	9.6557	—	7.899	11.5	68.4	5.8355
Alcohol_oil_molar_ratio	9	12	18	12	18	8	12
Reaction_temp (°C)	40	75	65	110	65	65	65
Reaction_time (min)	60	60	200	180	240	150	120
Catalyst amount (wt%)	3	6	4	0.5	3	5	4
Mixing_intensity (rpm)	600	—	—	400	600	—	—
Yield (%)	92.23	96.5	98.83	77.67	96.47	93.4	98.2
Density (kg m^−3^)	869	915	872	883	841	875	870
Viscosity (mm^2^ s^−1^)	3.26	11.2	4.1	5.34	4.2	3.89	4
Flash_point (°C)	—	168	162	167	73	142	149.5
Cloud_point (°C)	−7	—	1	11	−1	—	10
Pour_point (°C)	0.16	—	−5	9	−5	—	12
Acid_value (mg_KOH_ g^−1^)	—	0.3124	0.25	0.43	—	0.3	0.24
Cetane_number	—	58	57.64	—	—	—	50

The studies summarized in Table 11 differ simultaneously in feedstock composition, catalyst chemistry, metal loading, pore characteristics, catalyst dosage, alcohol-to-oil ratio, reaction temperature, and reaction time. The compiled data therefore do not support a direct causal relationship between BET surface area and reaction severity. Surface area should instead be interpreted as a potential contributing factor together with pore accessibility, active-site chemistry, metal dispersion/loading, and operating conditions. Likewise, the apparent performance differences among single- and mixed-metal systems should not be attributed to metal composition alone without controlled comparisons. Thus, Table 11 illustrates reported catalyst–feedstock–process combinations associated with high yields rather than establishing universal superiority, robustness, or scalability.

Fuel properties of the produced biodiesel largely met standard specifications for density, viscosity, and acid value. In contrast, cold flow properties varied mainly with feedstock type rather than catalyst, indicating that fatty acid composition remains the dominant factor controlling low-temperature behavior. Overall, the table demonstrates that optimized biochar-supported catalysts can effectively process waste-derived feedstocks while delivering high-quality biodiesel, reinforcing their suitability for sustainable and scalable biodiesel production.

### 4.8 Modeling of biodiesel production

Modeling of biodiesel production plays a central role in understanding reaction mechanisms, guiding process design, and enabling reliable scale-up and optimization. Conventional biodiesel models are predominantly based on first-principles reaction engineering, where mechanistic kinetic expressions are coupled with reactor mass and energy balances, phase equilibrium descriptions, and transport phenomena.^2,10^ Such physics-based frameworks provide high interpretability and predictive reliability, making them essential for reactor design, process intensification, and downstream separation development.^69,70^

Recent advances in biodiesel modeling focus on improved kinetic formulations for heterogeneous catalysis, detailed reactor representations including membrane-assisted packed beds, accurate thermodynamic treatment of partially miscible multicomponent systems, and systematic experiment-informed parameter estimation using statistically designed experiments. Collectively, these developments aim to enhance model fidelity while maintaining computational tractability, enabling effective integration into process simulation, optimization, and control applications.^94^

#### 4.8.1 Conventional models

Conventional biodiesel production models rely on first-principles reaction engineering, integrating mechanistic kinetics with reactor frameworks (batch reactors, continuous stirred-tank reactors (CSTRs), plug-flow reactors (PFRs), and packed beds), phase equilibria, and transport relations, followed by calibration using Arrhenius/Eyring parameters and experimental validation. These models remain essential for design, optimization, and scale-up due to their interpretability and predictive reliability.^2,10^ Recent advances are grouped into five categories and discussed below:

##### 4.8.1.1 Kinetic modeling: pseudo-homogeneous *vs.* heterogeneous reaction mixture

For base- or acid-catalyzed transesterification of triglycerides (TG) with methanol (MeOH) to produce fatty acid methyl esters and glycerol (Gly), conventional models often use a stepwise scheme (TG → DG → MG → Gly), with each step generating FAME. In a pseudo-homogeneous approach, the rate of the *i*th step can be expressed using a power law:1
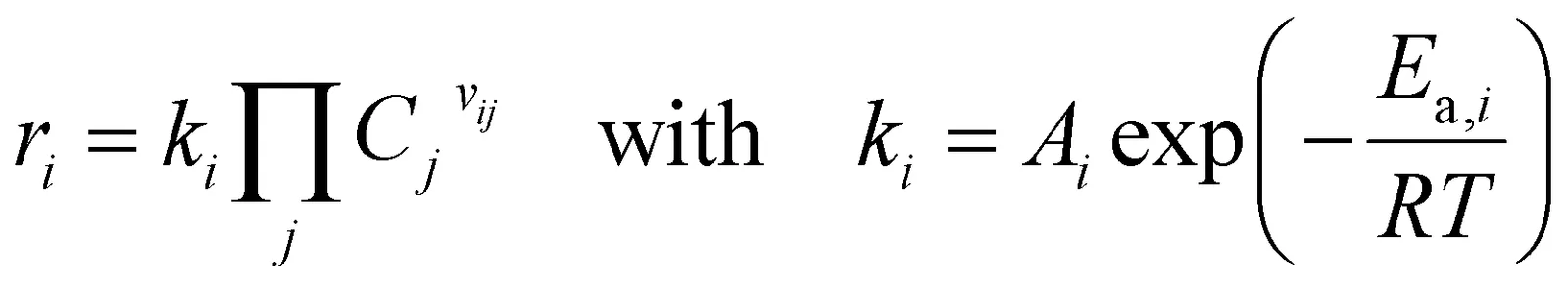
where *r*_*i*_ is the rate of reaction *i*, *k*_*i*_ is the corresponding rate constant, *C*_*j*_ is the concentration (or partial pressure) of species *j*, *ν*_*ij*_ is its reaction order in reaction *i*, *A*_*i*_ is the pre-exponential factor, *E*_a,*i*_ is the activation energy, *R* is the universal gas constant, and *T* is the absolute temperature.

For heterogeneous catalysts, Langmuir–Hinshelwood–Hougen–Watson (LHHW) or Eley–Rideal (ER) formulations provide more accurate descriptions. A typical ER expression for TG adsorbed on the catalyst surface reacting with dissolved MeOH is:2
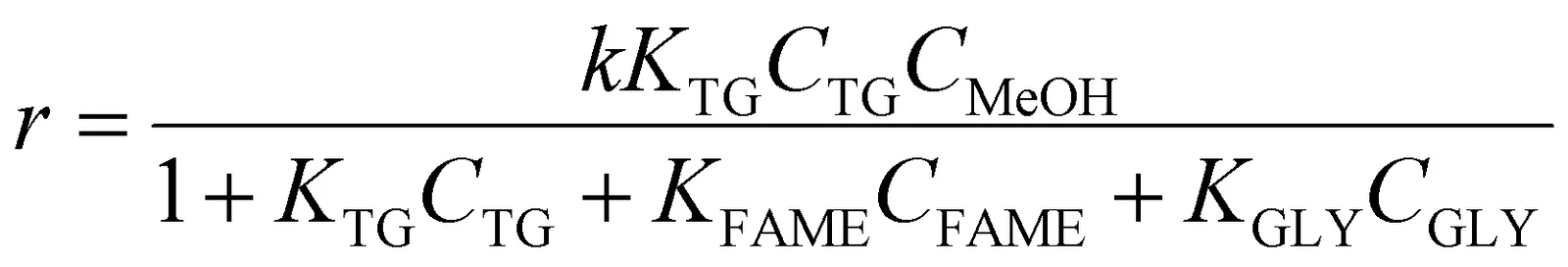
where *r* is the reaction rate, *k* is the kinetic rate constant, *K*_TG_, *K*_FAME_, and *K*_GLY_ are adsorption equilibrium constants for triglyceride (TG), fatty acid methyl ester (FAME), and glycerol (GLY), respectively, *C*_TG_, *C*_MeOH_, *C*_FAME_, and *C*_GLY_ are the corresponding species concentrations. The denominator accounts for competitive adsorption and site coverage by reactants and products.

Recent studies (2024–2025) increasingly adopt ER/LHHW models with sensitivity analyses to capture site competition, product inhibition, and to decouple kinetic from transport effects.^20,80^ Yao *et al.* (2024) present workflows for kinetic parameter determination, using integration/differentiation approaches and transient microreactor experiments coupled with in-line analytics, enabling robust Arrhenius/Eyring fits and identifiability assessments.^95^ Pacheco *et al.*^94^ advocate tiered kinetic models—from simplified power-law to mechanism-based—selected according to data availability, facilitating plant-wide simulations and surrogate optimization.

For temperature dependence, most studies still employ the Arrhenius equation, though some report Eyring–Polanyi formulations:3
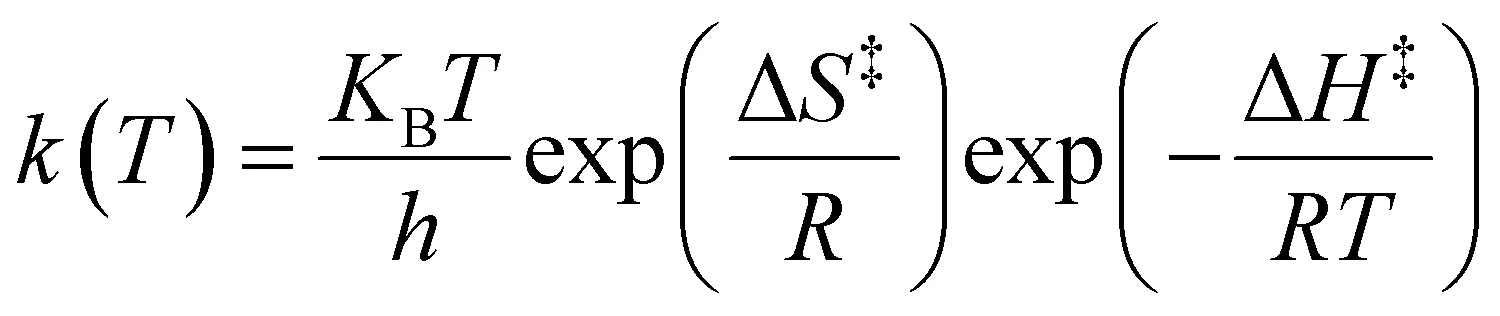
where *k*(*T*) is the temperature-dependent rate constant, *k*_B_ is the Boltzmann constant, *h* is Planck's constant, *T* is the absolute temperature, Δ*S*^‡^ is the activation entropy, Δ*H*^‡^ is the activation enthalpy, and *R* is the universal gas constant. This approach can reduce parameter correlation and improve extrapolation to elevated or supercritical conditions. Recent biodiesel research increasingly combines Eyring and Arrhenius analyses for more robust kinetic and thermodynamic characterization.^96^

##### 4.8.1.2 Reactor modeling: batch/CSTR/plug-flow and membrane-assisted packed beds

###### 4.8.1.2.1 Ideal reactors

Batch and CSTR models are commonly used for lab-scale kinetic studies and control-oriented analyses, linking mass balances to kinetic rate laws. For continuous production, plug-flow or packed-bed models are employed, incorporating effectiveness factors to account for intraparticle diffusion and Sherwood/Colburn correlations for external mass transfer, with energy balances capturing temperature effects, as transesterification can be mildly endothermic or exothermic depending on conditions.^97^

###### 4.8.1.2.2 Membrane-assisted packed beds (PBMR)

A 2-D heterogeneous model, accounting for mass and momentum balances across the catalytic bed and membrane with selective methanol permeation, showed that PBMRs can achieve ∼99.9% TG conversion at much shorter reactor lengths than FBRs (∼0.86 m *vs.* 2.75 m) under similar conditions. The study also reported a lower power demand (1313 *vs.* 1352 kW for an 8000 ton per year case), demonstrating process-intensification potential. These models resolve axial concentration and temperature profiles as well as radial gradients induced by the membrane and are solved numerically using ER kinetics.^95^

###### 4.8.1.2.3 Supercritical methanol (non-catalytic)

For high-temperature, high-pressure supercritical methanol (SC–MeOH) processes, conventional models use apparent, often second-order kinetics with strong temperature dependence and single-phase assumptions. Yu *et al.*^98^ updated SC-MeOH transesterification kinetics for rapeseed-oil surrogates and incorporated them into process simulations for design purposes.

##### 4.8.1.3 Phase equilibrium & thermodynamic modeling

Accurate liquid–liquid equilibrium (LLE) modeling is essential in transesterification and downstream separation due to the presence of partially miscible mixtures (FAME–Gly with MeOH partitioning). Recent studies on waste-oil biodiesel + methanol + glycerol and biodiesel + glycerol + triglyceride systems (303–333 K) correlated experimental data using NRTL and UNIQUAC, with NRTL outperforming UNIQUAC for WOBD–MeOH–Gly (RMSD ≈ 2.8% *vs.* 3.6%).^99^ Yang *et al.*^99^ also quantified how biodiesel enhances TG–MeOH mutual solubility, improving phase-behavior predictions during reaction and washing. These parameters are incorporated into process simulators such as Aspen and HYSYS, together with kinetic reactor models, to design decanters, extractive units, or hybrid separation processes (*e.g.*, membrane–flash systems).

##### 4.8.1.4 Experiment-informed parameter estimation and design of experiments

Conventional workflows combine designed experiments with nonlinear regression (least-squares or maximum likelihood) to estimate kinetic and adsorption parameters. Response surface methodology (RSM) is commonly employed to evaluate and optimize factors such as methanol-to-oil ratio, reaction temperature, catalyst concentration, and reaction duration, while also generating data for kinetic studies. Recent 2024–2025 studies, including microwave-assisted Li–CaO catalysts and WCO routes, report ≥90% yields and ANOVA-supported surfaces highlighting input interactions that enhance kinetic identifiability and reactor-scale prediction robustness. Johari *et al.*^100^ also summarized RSM's role in biodiesel process optimization.

##### 4.8.1.5 Model reduction and uncertainty

For plant-level optimization and control, detailed ER/LHHW kinetics are often simplified to power-law or quasi-homogeneous surrogates, using piecewise-isothermal schemes or parameter lumping. Pacheco *et al.*^94^ proposed selecting the simplest kinetic form that maintains fidelity within the operating window, aiding integration into mixed-integer linear/nonlinear programming (MILP/NLP) optimizers or real-time model predictive control (MPC) frameworks. Standard practice also includes local and global sensitivity analyses to assess parameter identifiability and Monte Carlo simulations to propagate uncertainties in kinetics and LLE, thereby bounding conversion and purity predictions.

#### 4.8.2 Machine learning models in biodiesel production

This section reviews machine-learning methods used in biodiesel research for yield prediction, process optimization, and fuel–property estimation. Although these models often show strong predictive performance, most rely mainly on feedstock and operating variables, with limited inclusion of catalyst-specific features. This is a key limitation for metal-doped activated-carbon catalysts, where properties such as surface area, pore structure, acidity/basicity, metal identity, dispersion, and loading strongly influence performance. Thus, the key question is not only whether ML can predict biodiesel yield, but whether it can learn transferable structure–property–process–performance relationships for metal-doped AC systems.^12,21^

##### 4.8.2.1 Current use and scope in biodiesel studies

The review focuses on ML studies relevant to catalyst selection, reaction optimization, and catalyst-aware prediction. In biodiesel research, ML is mainly used to link operating conditions and feedstock features to yield and fuel properties, while catalyst descriptors remain underrepresented. Thus, the key value of ML is enabling interpretable, transferable relationships between catalyst structure, composition, and performance.^21^Fig. 9 classifies ML into supervised learning (classification/regression), unsupervised learning (clustering), and reinforcement learning (interactive optimization).

**Fig. 9:**
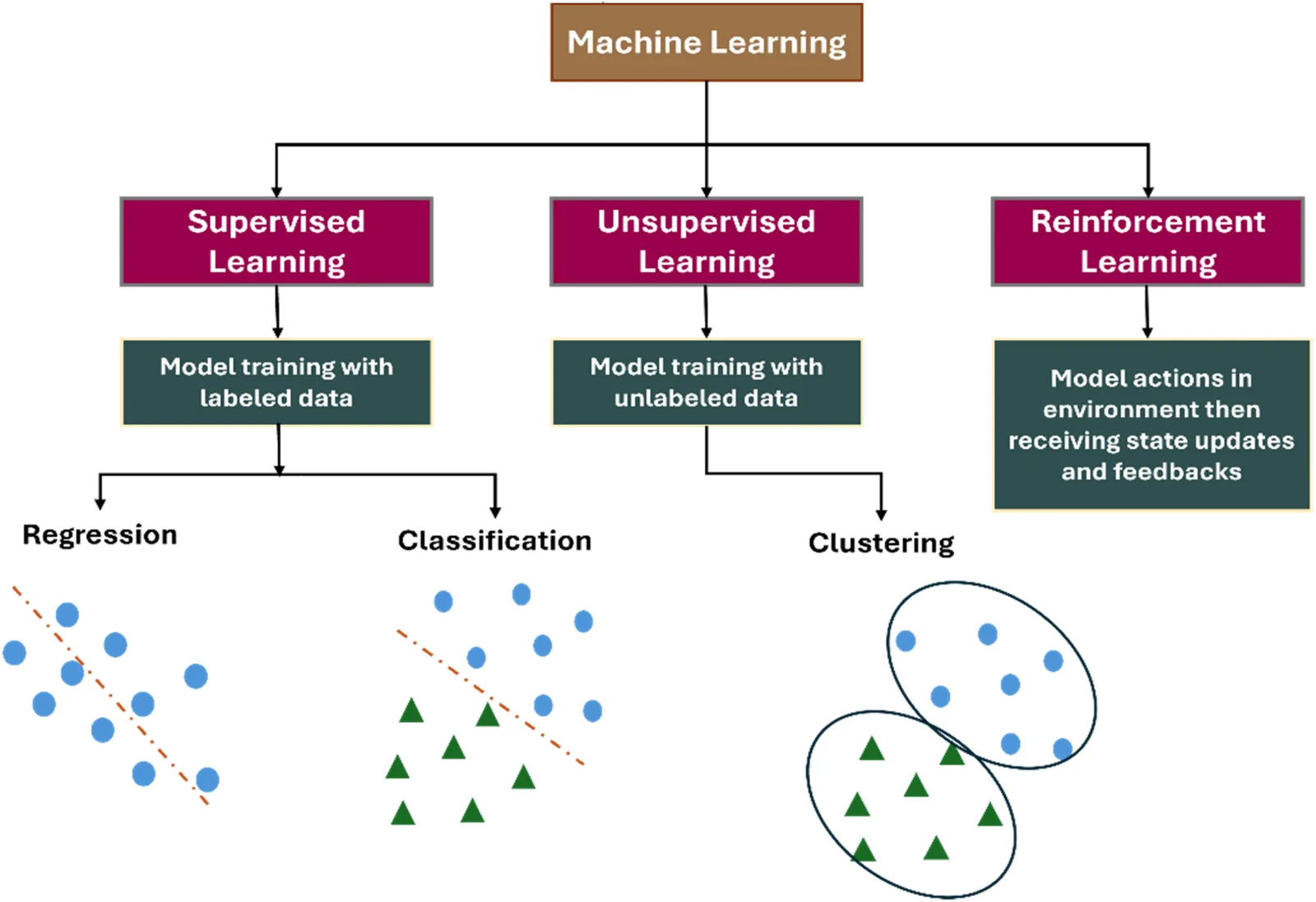
The main types of machine learning algorithms.

A typical ML workflow in chemical engineering includes problem formulation, data collection, preprocessing, model training, and testing (Fig. 10). In biodiesel applications, ML is used to optimize feedstock selection, catalyst performance, and reaction conditions (*e.g.*, temperature, methanol-to-oil ratio, and catalyst loading) using lipid profiles, kinetics, and FTIR/NIR data to predict yield and conversion efficiency. Chemometric models further evaluate fuel quality (viscosity, cetane number, and stability) and process performance across production stages. However, key limitations remain, including small datasets, overfitting, limited generalization, and lack of external validation.^101^

**Fig. 10:**
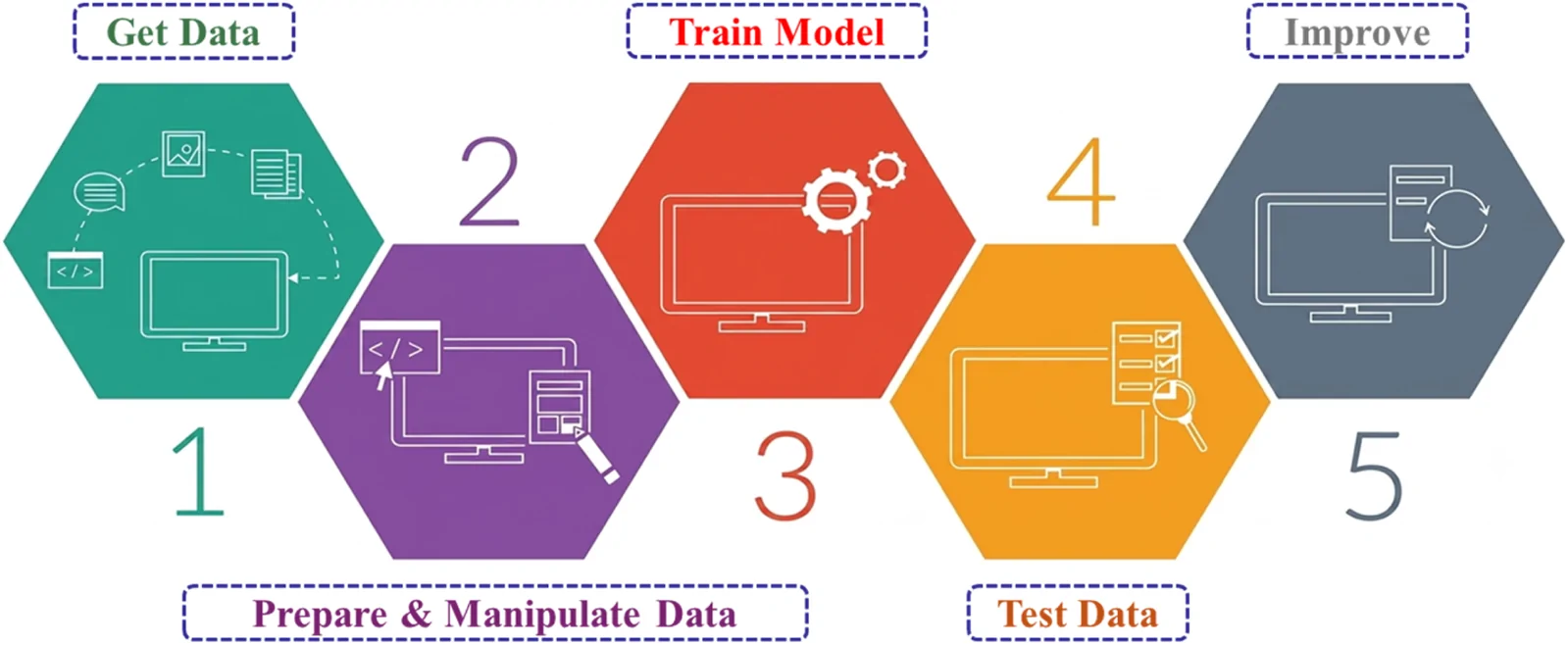
Workflow of a machine learning model.

##### 4.8.2.2 Catalyst-aware ML applications for carbon-based biodiesel catalysts

Within the literature identified in this review, direct ML applications that explicitly incorporate carbon-catalyst characteristics remain limited compared with general ML studies for biodiesel prediction and process optimization. Ebrahim *et al.*^102^ presented a catalyst-aware explainable ML framework for biodiesel-yield prediction involving metal-doped biochar and activated-carbon catalysts, making this study directly relevant to the catalyst–ML integration considered in this review. Ohale *et al.*^103^ also reported an ML-informed one-pot biodiesel-synthesis study using a magnetic sulfonated biobased catalyst, illustrating the potential of combining catalyst-related information with operating conditions in data-driven process analysis. In contrast, most studies summarized in the broader ML literature rely primarily on reaction conditions, feedstock characteristics, product composition, or spectroscopic information as model inputs.^104,105^ As summarized in Table 12, the mentioned studies primarily predict biodiesel properties and FAME yield or conversion using neural-network, neuro-fuzzy, and support-vector-based models, generally with high prediction accuracy.

**Table 12:** Applications of machine learning techniques in biodiesel production and property prediction

Application type	Target (output)	Inputs (main variables)	ML model	Dataset size	Model evaluation	Key findings	Ref.
Property prediction	Density, KV, water %, methanol %	NIRS data	MLPNN	103	RMSE ≈ 0.068–0.51	Accurate modeling	120
	KV, IV, OSI	13 FAMEs	MLPNN	98	*R* ≈ 0.99	Good OSI prediction	121
	KV, PP, CP, IV	Temp., FA %, molar mass	ANFIS	25–59	*R* ^2^ ≈ 0.94–0.996	Strong nonlinear modeling	126
	KV, PP, CP, IV	FAME profile	PSO-LSSVM	25–59	*R* ^2^ ≈ 0.996–0.9999	Better than ANFIS	127
Cold flow prediction	CP, PP, CFPP	9 FAMEs	MLPNN	103	*R* ^2^ ≈ 0.94–0.98	Reliable cold flow estimation	122
Enzymatic process modeling	FAME yield, exergy efficiency	MeOH ratio, enzyme conc., time	MLPNN	27	*R* ^2^ ≈ 0.99	Better than RSM	123
	FAME yield	Time, *T*, MeOH ratio	MLPNN	30	*R* ^2^ ≈ 0.97	Outperformed RSM	124
Enzymatic yield prediction	FAME yield	Time, enzyme %, *T*	MLPNN	35	*R* ^2^ ≈ 0.996	Very high accuracy	125
Mechanically assisted process	FAME yield	Catalyst %, MeOH ratio, *T*	MLPNN	27	MSE ≈ 0.048	Scaled conjugate gradient (SCG) best algorithm	129
Conversion modeling	FAME yield	Catalyst %, MeOH ratio, *T*	MLPNN	9	APE ≈ 0.23–0.83	Good conversion estimation	130
Yield simulation	FAME yield	*T*, time, catalyst %, oil ratio	MLPNN	45	Not reported	Acceptable prediction	131
Yield modeling	FAME yield	Catalyst %, MeOH ratio, time	MLPNN	29	*R* ^2^ ≈ 0.99	Accurate yield model	132
FAME content prediction	FAME yield	MeOH ratio, catalyst %, *T*	MLPNN	156	*R* ≈ 0.95	Large dataset study	133
Process optimization	FAME yield	*T*, catalyst %, time, MeOH ratio	MLPNN-GA	21	*R* ^2^ ≈ 0.99	GA improved prediction	128
Model comparison	FAME yield	MeOH ratio, catalyst %, time	MLPNN	17	*R* ^2^ ≈ 0.99	MLPNN > LR	134
Model comparison	FAME yield	Catalyst %, MeOH ratio, *T*	MLPNN	29	*R* ^2^ ≈ 0.997	MLPNN > RSM	135

For waste-derived activated-carbon and biochar catalysts, a major limitation is therefore the limited and non-standardized representation of catalyst descriptors. A catalyst-aware dataset should link precursor and activation route, carbonization or calcination conditions, metal identity and loading, BET surface area, pore characteristics, acid/base functionality, metal phase or dispersion where available, and catalyst recoverability with feedstock characteristics, reaction conditions, biodiesel yield and fuel properties, reuse-cycle performance, and metal leaching. Incorporating these variables would allow ML to move beyond process-only prediction toward catalyst screening and learning structure–property–process–performance relationships.

Given the limited number of directly catalyst-aware studies identified and the heterogeneity of their datasets and descriptors, cross-study comparison of ML algorithms specifically for waste-derived carbon catalysts is not yet justified. The current evidence instead indicates a need for standardized catalyst–process–performance datasets, consistent descriptor reporting, external validation, and experimentally validated ML-guided catalyst screening and optimization.

##### 4.8.2.3 Common machine learning models in biodiesel production

ML enhances biodiesel production by enabling data-driven process optimization, quality control, and predictive decision-making. ML models uncover patterns in production data, allowing accurate predictions without explicit programming. Common approaches include supervised learning for predicting feedstock composition and assessing biodiesel quality,^94,106^ unsupervised learning for clustering and anomaly detection in process data, and reinforcement learning for real-time optimization of parameters such as temperature, catalyst dosage, and reaction time.^106,107^

A critical step in applying ML to biodiesel production is feature selection and data preprocessing, including the identification of relevant inputs (*e.g.*, fatty acid content, viscosity, and temperature) and the treatment of missing values, outliers, and scaling to improve model accuracy. Algorithms such as decision trees, random forests, *k*-nearest neighbours, and neural networks have been applied to predict optimal feedstock composition and to model and optimize transesterification reactions.^98,101^ They are also used to monitor and control process parameters in real time and to predict key quality metrics, such as viscosity, cetane number, and oxidative stability, while detecting anomalies.^22,108^ These approaches enhance yield, quality, and process stability, reduce resource use and costs, and benefit from continuous learning to adapt to new data and changing conditions, thereby maintaining predictive reliability.^22,23^ Accordingly, the following section reviews and compares the main machine-learning models employed in biodiesel production, highlighting their underlying principles, predictive capabilities, and suitability for process optimization and quality assessment.

###### 4.8.2.3.1 Artificial neural networks

Artificial Neural Networks (ANNs), inspired by mathematical neurobiology, are well-suited for modeling complex, nonlinear systems such as biodiesel production. Unlike mechanistic models, ANNs can handle uncertain, incomplete, and noisy data without requiring detailed knowledge of the underlying physical processes, relying instead on observed input–output relationships.^24^Fig. 11 illustrates a schematic diagram of the artificial neural network structure. ANNs mimic the brain's neural architecture, where dendrites receive signals, the soma integrates them, and the axon transmits outputs through synapses when a threshold is exceeded. In artificial networks, neurons receive inputs [*X*_1_, *X*_2_,…, *X*_*m*_], which are multiplied by weights (*W*_k*j*_), summed with a bias (*b*_k_), and passed through an activation function, such as the sigmoid or tanh, to generate outputs (*y*_k_).^109,110^

**Fig. 11:**
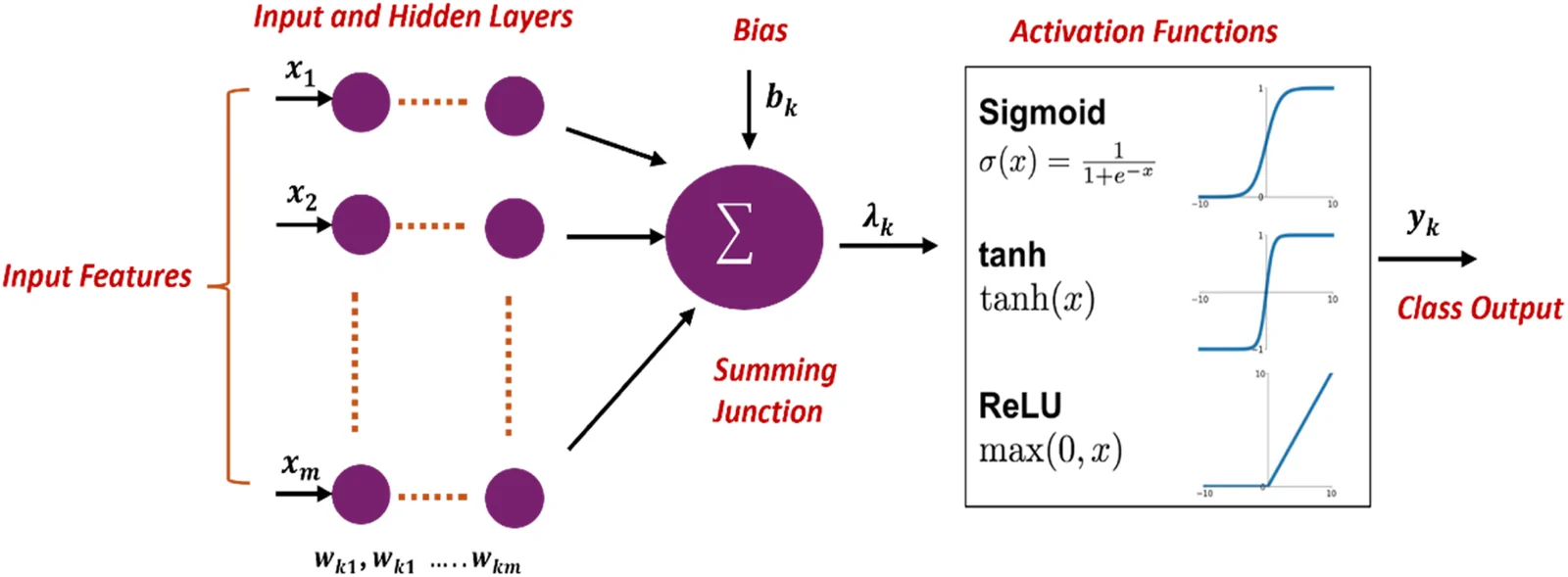
Schematic diagram showing the artificial neural network structure.

ANNs are widely used in biodiesel research to predict yield, ester content, and fuel properties under varying feedstocks, catalysts, and operating conditions. They also model key process variables (temperature, alcohol-to-oil ratio, catalyst loading, time, and mixing) to support optimization of transesterification and esterification. Overall, ANNs effectively capture nonlinear relationships in complex, noisy datasets, enabling accurate prediction, sensitivity analysis, and process optimization.^25^

However, despite their predictive strength, ANNs are often criticized as black-box models because their internal decision-making process lacks direct physical meaning and is difficult to interpret in terms of reaction mechanisms or transport phenomena. This limitation reduces their ability to provide mechanistic insight and may reduce confidence when extrapolating beyond the training domain. Such limitations can be addressed by physics-informed neural networks (PINNs), which enhance conventional ANN frameworks by embedding governing physical laws and kinetic constraints into the learning process, thereby improving physical consistency, interpretability, and generalization.

###### 4.8.2.3.2 Physics-informed neural networks

In recent decades, computational advances have transformed the study of complex systems beyond traditional theory-experiment approaches. Differential equations (ODEs/PDEs) remain central for modeling phenomena such as heat transfer, fluid dynamics, and reaction kinetics in chemical and energy processes. Numerical methods like the finite element method (FEM), which is a numerical technique used to solve partial differential equations by discretizing a complex domain into smaller elements, enable the simulation of transport phenomena such as heat transfer, fluid flow, and reaction kinetics in engineering systems, and particle-based approaches approximate PDE solutions but face high computational costs and challenges with multi-scale, nonlinear, or high-dimensional problems. Physics-informed machine learning (PIML) models integrate physical laws with data-driven models, improving generalization and enabling accurate predictions even with few or incomplete experimental data.^111^

PINNs are a leading PIML framework that embeds PDEs, boundary, and initial conditions directly into the neural network loss function, minimizing both data errors and PDE residuals to ensure physically consistent solutions. Fig. 12 illustrates the general framework of the PINN method. This mesh-free approach effectively handles forward and inverse PDE problems, complex geometries, sparse data, and coupled nonlinear dynamics. In biodiesel research, PINNs enable accurate modeling of fluid flow, transport phenomena, and reaction kinetics, integrating experimental data with first-principles knowledge to address challenges in sustainable fuel production.^111^

**Fig. 12:**
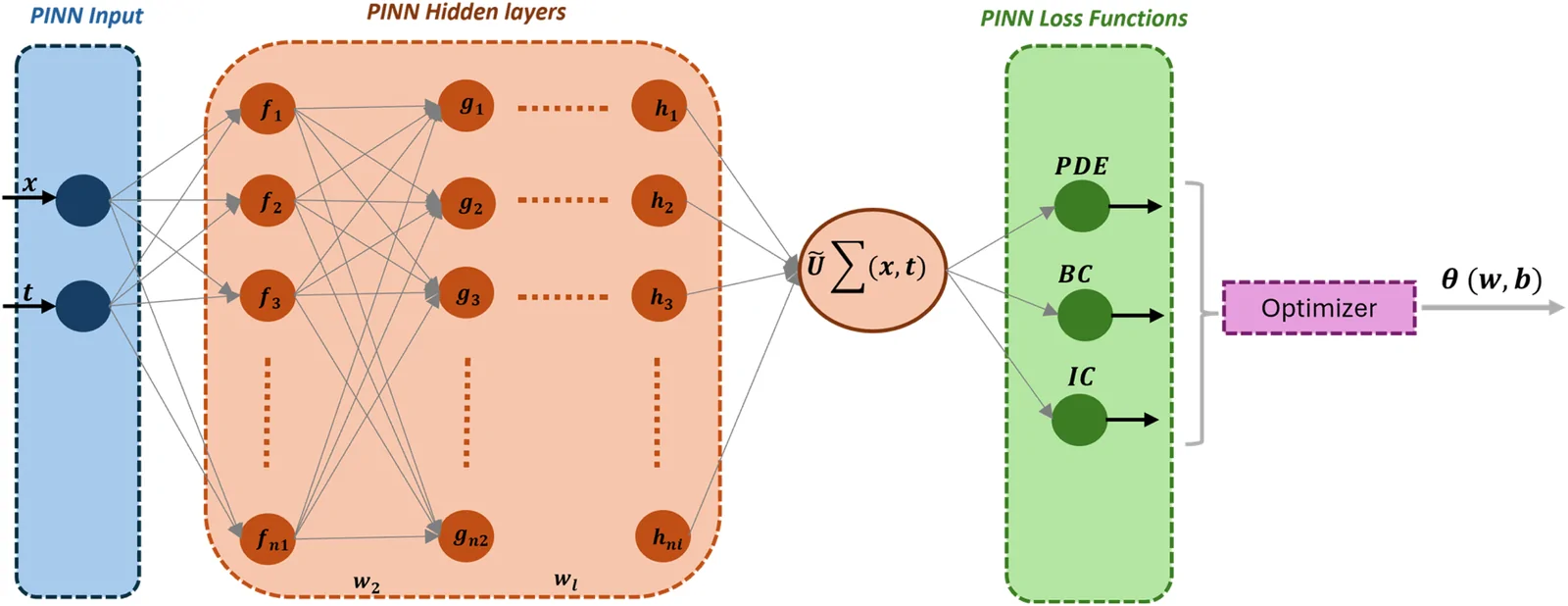
General framework of physics-informed neural networks.

Understanding PINNs requires summarizing the key equations that define their structure and performance. PINNs are deep learning models that embed governing physical equations, such as ODEs, into their loss functions, enabling predictions that are both accurate and physically consistent. Unlike conventional ML models, they enforce governing equations, such as mass or energy balances, enabling solutions even with limited data. In biodiesel production, the Bio-PINN project at Polytechnique Montréal applied PINNs to identify kinetic parameters for canola-oil transesterification in a microwave-assisted batch reactor using a mass-balance and generalized power-law rate formulation:^112^4
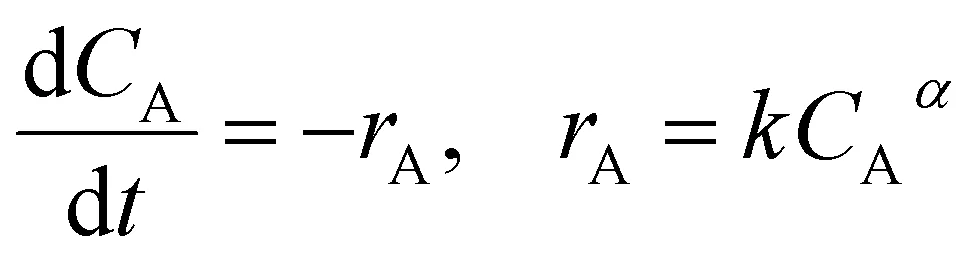
here, *C*_A_ denotes the reactant (glyceride) concentration, *r*_A_ the consumption rate, and *k* and *α* are the unknown reaction rate constant and order, respectively, inferred by the PINN. Thus, the formulation is not restricted *a priori* to first-order kinetics. The network is trained to predict the concentration profile *C*_A_(*t*) while simultaneously estimating *k* and *α*.

Experimental data were collected from a microwave reactor, with glyceride concentrations measured *via* Gas Chromatography–Flame Ionization Detection (GC-FID) and temperature recorded using an infrared sensor. The trained network predicts concentration profiles and infers kinetic parameters, validated against numerical solutions from classical solvers (*e.g.*, Runge–Kutta) and benchmarked against conventional nonlinear regression using the Trust Region Reflective (TRF) algorithm. An artificial dataset simulating a reversible reaction sequence A ⇌ B + C followed by C ⇌ D was also used to test robustness. Results show that PINNs effectively integrate domain knowledge with data-driven learning, improving predictive accuracy and reducing experimental effort, making them a powerful tool for parameter identification in complex processes such as microwave-assisted transesterification.

###### 4.8.2.3.3 Support vector machines

Support Vector Machines (SVMs) are supervised algorithms primarily used for classification, as depicted in Fig. 13, while they can also be applied to regression tasks. They identify an optimal hyperplane that separates classes with the maximum margin, determined by a few critical training points called support vectors, which enhances generalization and reduces overfitting even with limited data.^113^ These properties have made SVMs widely applied in chemical engineering problems, particularly in biodiesel production, where they have been used to predict biodiesel yield, ester content, and key fuel properties under varying feedstock characteristics, catalyst types, and reaction conditions.^25^

**Fig. 13:**
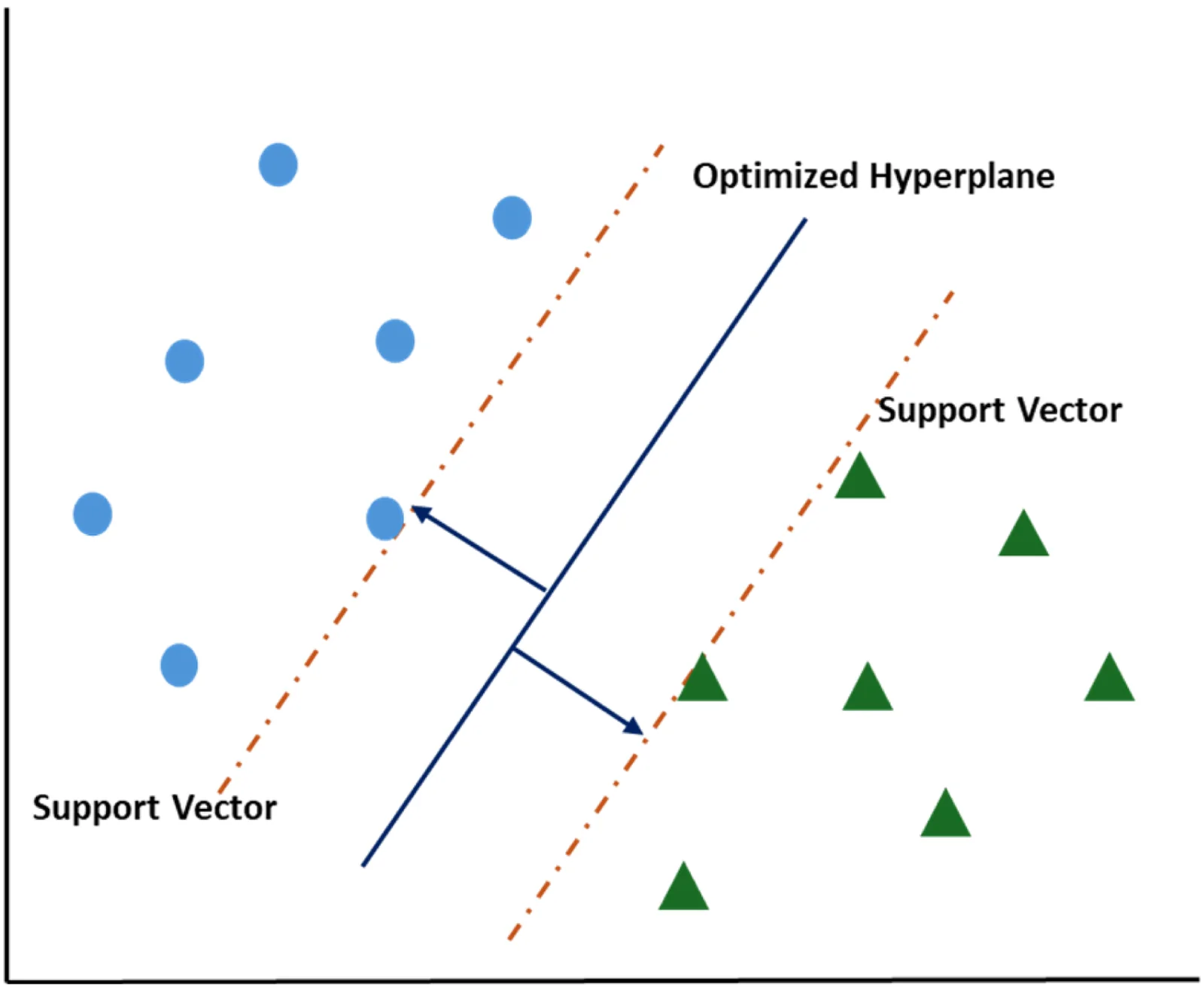
Illustration of support vectors and optimal separating hyperplane.

In biodiesel research, SVMs have proven to be reliable tools for modeling complex nonlinear relationships, especially when process variables are carefully selected and kernel functions are appropriately defined, enabling accurate prediction and optimization of transesterification and esterification performance.^25^ As nonlinear, kernel-based models, they produce sparse solutions that rely on a subset of training points called support vectors and are recognized for their universal function approximation capability.^104^ The SVM constructs a predictive function *y*(*x*) (eqn (5)) designed to minimize overall prediction error.

5 *y*(*x*) = *w*^T^·*∅*(*x*) + *b*

here, *w* is the weight vector defining the model, *b* is the bias term, and *∅*(*x*) is a mapping function that converts input data into a higher-dimensional feature space. Unlike traditional linear regression, where the cost function is based on squared errors (eqn (6)), SVM uses the *ε*-insensitive loss function (eqn (7)). This function ignores errors within a certain margin *ε* and penalizes only deviations that exceed this margin, thereby enhancing robustness to minor fluctuations.6
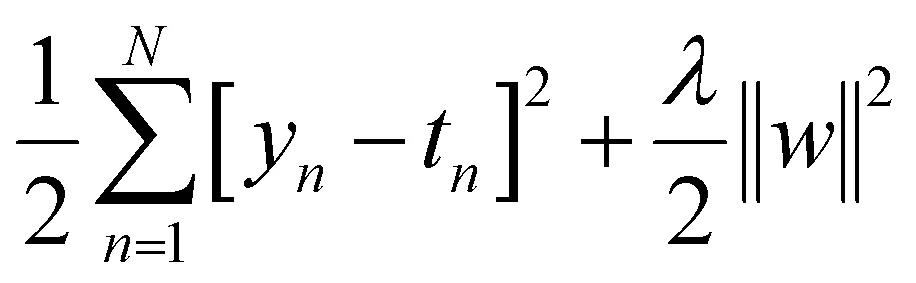
7
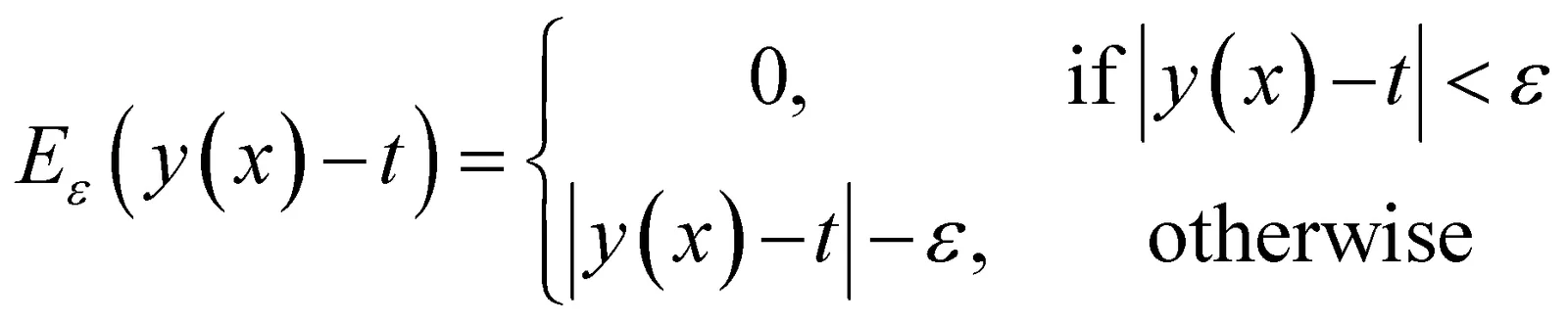


In eqn (6), *N* is the number of training samples, *y*_*n*_ is the predicted output for the *n*-th sample, *t*_*n*_ is the corresponding target (actual) value, ‖*w*‖^2^ is the squared Euclidean norm of the weight vector representing model complexity, and *λ* is the regularization parameter controlling the trade-off between fitting accuracy and model smoothness. In eqn (7), *ε* defines the width of the insensitive tube around the regression function within which no penalty is applied, and |*y*(*x*) – 1| represents the absolute prediction error.

The objective then becomes minimizing the cost function given in eqn (8), where the penalty term *C* controls the compromise between the complexity of models and tolerance to error.8
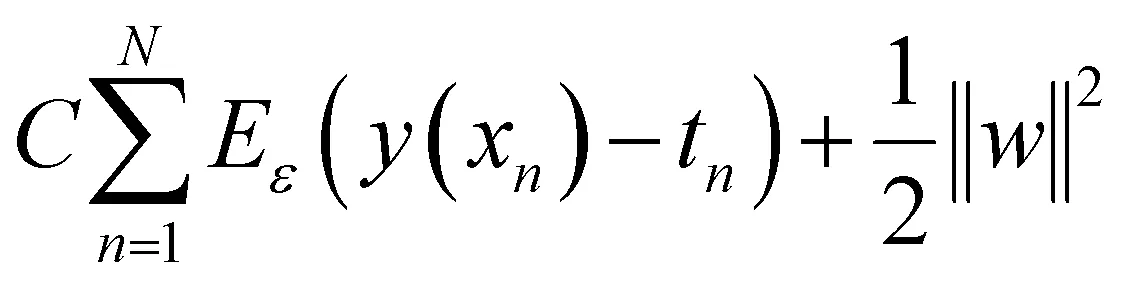


This leads to a more complex solution, expressed first in terms of inner products (eqn (9)), and later reformulated using a kernel function in eqn (10), where *α*_*i*_ and 
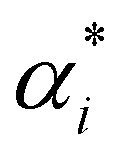
 are Lagrange multipliers obtained through optimization.9
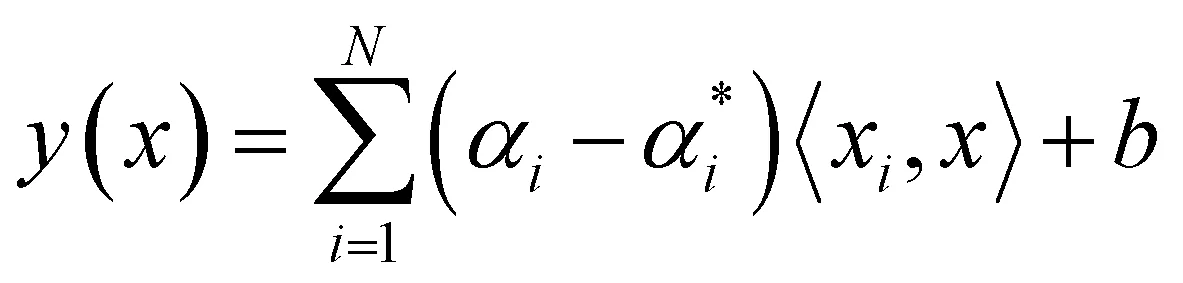
here, *α*_*i*_ and 
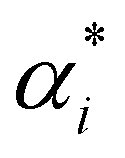
 are Lagrange multipliers obtained through quadratic optimization, *x*_*i*_ represents the *i*-th support vector, and 〈*x*_*i*_, *x*〉 denotes the inner product between training and test samples.10
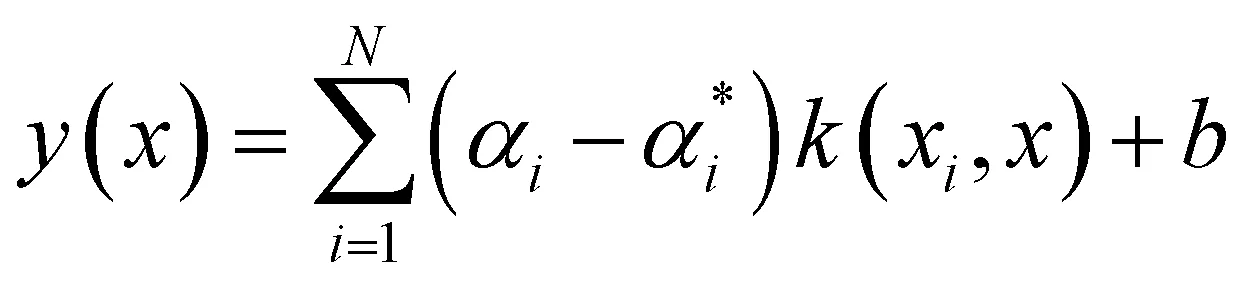


In eqn (10), *k*(*x*_i_, *x*) is the kernel function that implicitly computes the inner product in the transformed feature space without explicitly evaluating *ϕ*(*x*).

Three kernels are commonly utilized: linear (eqn (11)), polynomial (eqn (12)), and Gaussian RBF (eqn (13)), each transforming inputs into increasingly complex feature spaces.

11 *k*(*x*_*i*_, *x*) = *x*_*i*_^*T*^*x*

12 *k*(*x*_*i*_, *x*) = 〈*x*_*i*_·*x*^d^〉

13
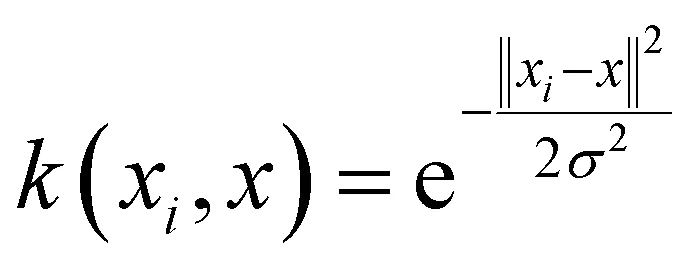
where *σ* is the kernel width parameter determining the spread of the Gaussian function, and ‖*x*_*i*_ – *x*‖^2^ is the squared Euclidean distance between samples.

The methodology, including kernel selection, model training, and evaluation, was implemented in ref. 104. SVMs were trained using 50 repetitions of 10-fold cross-validation on a dataset of 56 entries from a design of Experiments, providing robust generalization error estimates. Hyperparameters were fine-tuned in each iteration, with performance assessed *via* RMSE and correlations between predicted and observed values. The best SVMs were further validated using nine independent experiments to evaluate real-world generalization.

A hybrid SVM–Genetic Algorithm (GA) framework was developed for biodiesel production from waste cooking oil, using a Box–Behnken design to vary catalyst concentration, methanol-to-oil ratio, reaction time, stirring speed, and impurity levels. Experimental outputs, including yield, viscosity, turbidity, density, and heating value, were used to train several regression models. Among the linear regression and SVM models using linear, polynomial, and radial basis function (RBF) kernels, the SVM model with the radial basis function kernel achieved the highest accuracy for most outputs, whereas the polynomial kernel performed best for turbidity. When coupled with GA, the optimal SVM models identified operating conditions that maximized biodiesel yield and quality, including a catalyst dosage of approximately 1 wt%, along with optimal values for the other process variables.^104^

###### 4.8.2.3.4 Decision trees and random forest

Decision trees are supervised machine-learning models that recursively partition data according to the most informative splits in the input variables. In biodiesel systems, they can be applied to classify or predict biodiesel yield based on process conditions and catalyst descriptors. Variables such as catalyst loading and reaction temperature may be used to divide the data into low- and high-yield regions, as shown in Fig. 14. By selecting optimal splitting variables and thresholds during training, decision trees generate a transparent, rule-based structure that is straightforward to interpret. Their key contribution is the ability to reveal influential process parameters, extract simple decision pathways, and support interpretable analysis of biodiesel production behavior.^24^

**Fig. 14:**
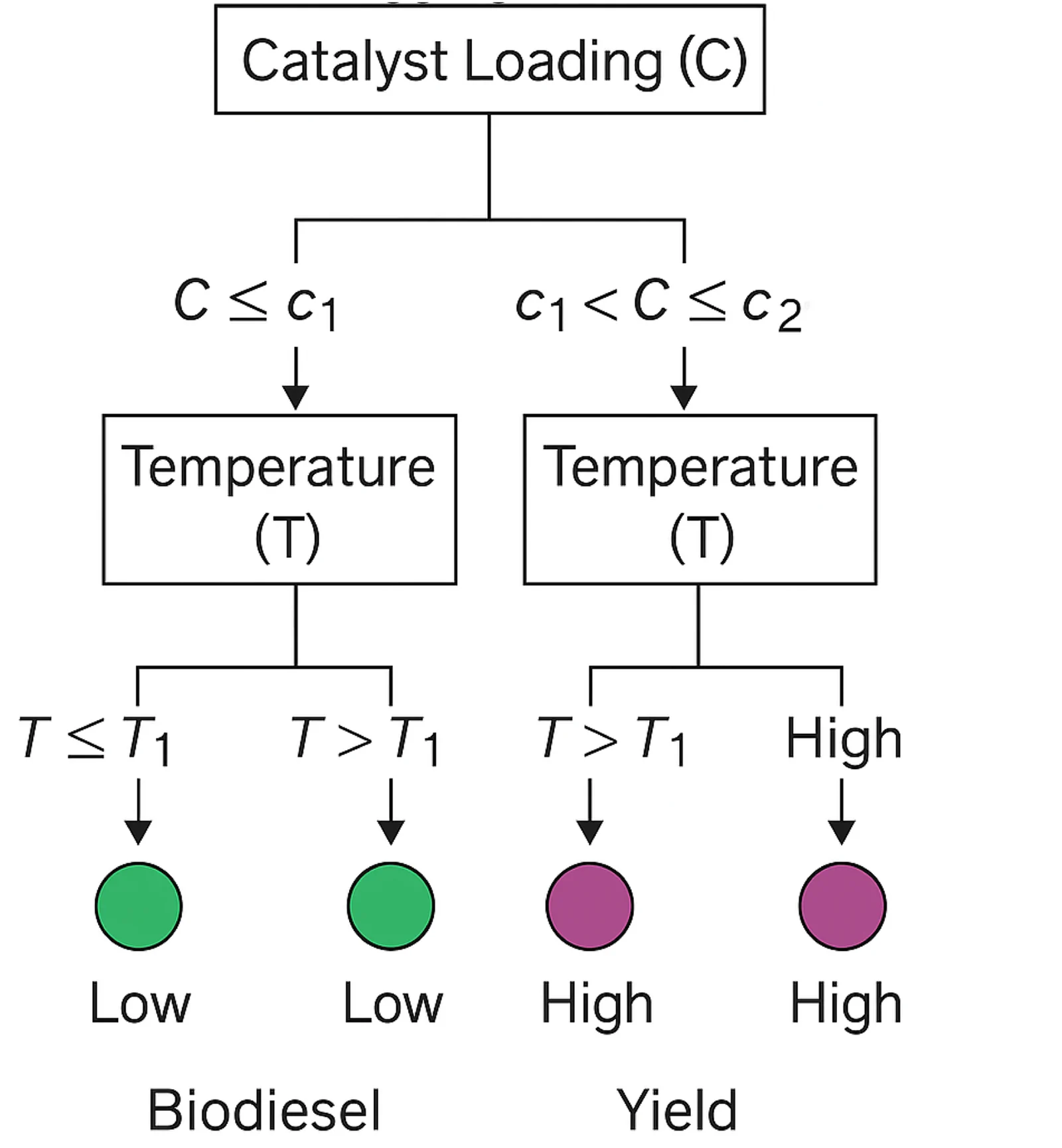
Training a decision tree and its resulting decision boundaries.

Decision trees are also robust to outliers and can accommodate both numerical and categorical inputs. Nevertheless, excessively deep trees are prone to overfitting and usually require pruning or other complexity-control strategies. While a single decision tree may offer lower predictive performance than more advanced machine-learning methods, it provides the conceptual foundation for ensemble models such as bagging and random forest algorithms.

Random forests are an ensemble learning method based on bagging, in which multiple decision trees are trained on bootstrapped datasets while a random subset of features is considered at each split, thereby increasing model diversity and improving predictive accuracy. As illustrated in Fig. 15, the random forest method involves four main steps: sampling the data, constructing individual decision trees, generating predictions from each tree, and aggregating these predictions to produce the final output. Random forests handle medium-to-large datasets with mixed data types and missing values, though interpretability is reduced, with feature importance scores helping identify key predictors.^114^

**Fig. 15:**
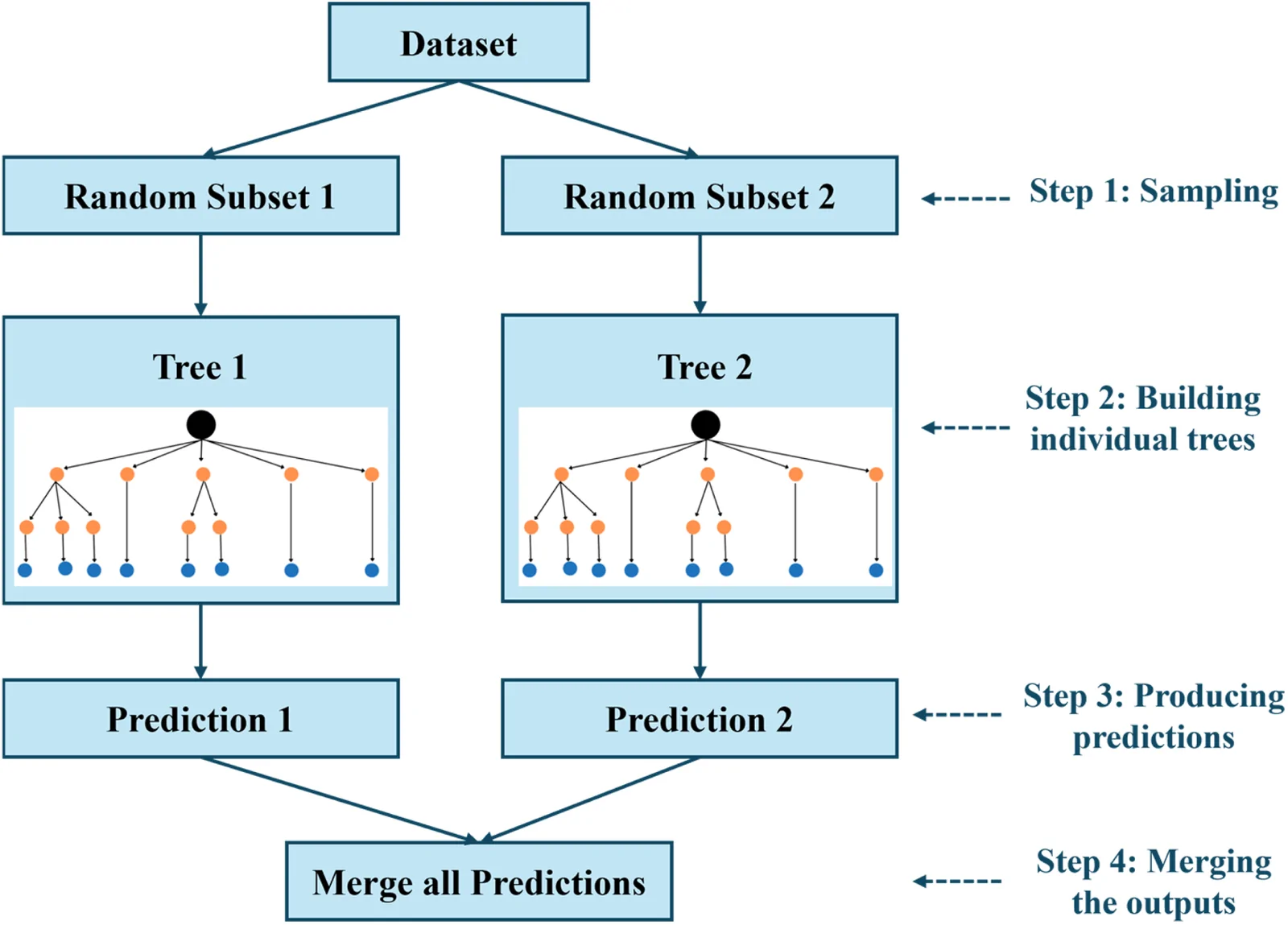
Illustration of the random forest procedure with multiple decision trees and aggregated prediction.

Random forest regression is a powerful ensemble technique that merges bagging with random feature selection. It builds multiple decision trees independently, each trained on a bootstrap sample of roughly two-thirds of the original dataset *D* containing *N* points.^26^ A total of *K* uncorrelated trees are trained, and while individual trees have low bias but high variance, averaging their predictions stabilizes and improves accuracy. The final prediction for an input *x* is the mean output across all *K* trees (eqn (14)):14
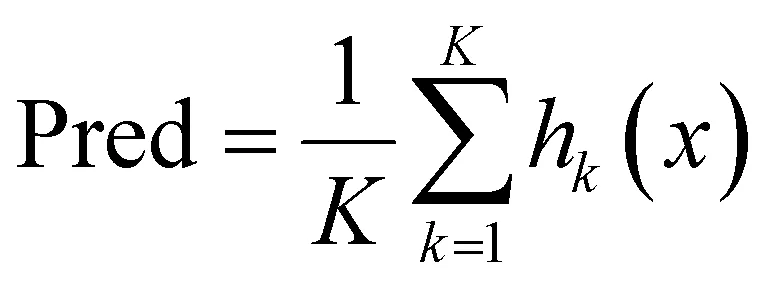
where (*K*) is the total number of trees and (*k*) is the tree index.

A major benefit of bagging is its capacity to lower prediction variance and prevent overfitting relative to individual decision trees. Performance is assessed using out-of-bag (OOB) samples, which are roughly one-third of the data left out from each tree's bootstrap sample. These OOB data serve as an internal validation set to estimate accuracy. The mean squared error (MSE) on OOB data is calculated using eqn (15), where *y*_*i*_ is the observed value and *ȳ*^OOB^_*i*_ is the mean prediction from all trees that did not include *y*_i_ in training.15
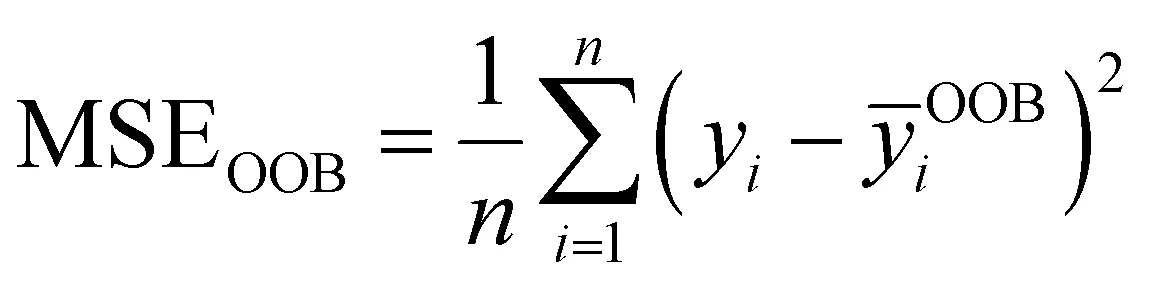


Subsequently, the OOB-based coefficient of determination *R*_OOB_^2^ is computed to assess model fit, defined in eqn (16) as:

16 *R*_OOB_^2^ = 1 − MSE_OOB_/Var_*y*_

here, Var_*y*_ denotes the total variance of the target variable across the dataset. This OOB estimation provides a reliable internal measure of model performance without requiring a separate validation set.

Random Tree (RT)-based methods are advantageous for modeling complex biodiesel systems because they can capture nonlinear behavior and higher-order interactions among feedstock characteristics, catalyst properties, and reaction conditions with little need for extensive data preprocessing. They are also computationally efficient, scalable, and capable of providing variable-importance information that helps identify influential predictors. These characteristics make them well-suited for heterogeneous regression tasks such as biodiesel yield or fuel–property prediction. Nevertheless, the increased randomness and multi-tree structure can reduce the direct interpretability available in a single decision tree, despite improving overall robustness and predictive stability.^115,116^

In biodiesel modeling, RT has demonstrated strong potential for predicting several important hydroprocessed biodiesel properties, such as oxidation stability, calorific value, kinematic viscosity, density, flash point, and acid value. Its key contribution is the ability to model complex nonlinear relationships and provide accurate simultaneous predictions of multiple fuel properties, making it useful for biodiesel quality assessment and process analysis.

Nevertheless, extremely high predictive accuracy should be interpreted carefully, as it may in some cases reflect overfitting rather than genuine generalization performance.^117^ RT's robustness was consistent across different FAME composition strategies (MFC, SCSF-DUm, LCSF-DU), whereas ANN performance depended on the method, with the best performance observed for MFC.^117^ Comparative analyses further confirmed RT's superiority over SVM, GA, MLR, and ANFIS models in terms of *R*^2^, RMSE, and MAPE, demonstrating its reliability and high predictive accuracy for predicting biodiesel fuel properties.

##### 4.8.2.4 Modeling process optimization

In biodiesel production, hyperparameter optimization is crucial for improving the performance of ML models in process modeling, control, and yield prediction. Advanced optimization techniques are applied to fine-tune model parameters, enhancing the accuracy and robustness of regression models for biodiesel-related applications.^21^

Grid search is a widely used, exhaustive method for hyperparameter tuning. While simple and interpretable, it can be computationally demanding for large parameter spaces and has been applied in biodiesel modeling to identify optimal configurations.^118^

Genetic Algorithms (GA) mimic evolutionary processes – selection, crossover, and mutation-to optimize hyperparameters, effectively exploring complex, non-linear search spaces in biodiesel modeling.^119^

Ant Colony Optimization (ACO), inspired by ant foraging, uses pheromone trails and heuristics to guide the search toward promising hyperparameter regions, effectively adapting to complex biodiesel models.^21^

Whale Optimization Algorithm (WOA) mimics humpback whale hunting strategies, balancing exploration and exploitation to robustly search hyperparameter spaces in biodiesel-related regression tasks.^115^

Bayesian Optimization Algorithm (BOA) uses a probabilistic surrogate, typically Gaussian processes, to iteratively guide the search, efficiently identifying optimal hyperparameters in biodiesel modeling under limited computational budgets.^21^

K-fold cross-validation (K-fold CV) partitions data into K subsets to assess model performance and ensure generalizability. Combined with BOA, it has been used to optimize ANN and SVR models for biodiesel prediction, enabling accurate hyperparameter tuning while reducing overfitting risk.^105^

These optimization strategies enable the development of accurate, generalizable ML models for predicting biodiesel yield, quality, and process efficiency. By automating hyperparameter selection and refining model performance, they advance data-driven process optimization. ML is increasingly applied to estimate biodiesel physicochemical properties, which are often costly or time-consuming to measure but are critical for engine performance and safety. Key targets include cetane number, viscosity, density, flash/fire point, heating value, cold-flow properties, iodine value, and oxidative stability. Models are trained on descriptors reflecting chemical and thermophysical behavior, including FAME profiles, process conditions (*e.g.*, temperature and pressure), spectroscopic signatures (NIRS/mid-FTIR), and engineered molecular descriptors.

ML has been widely applied in biodiesel production for both physicochemical property prediction and process optimization, as shown in Table 12. The most frequently predicted properties include KV (kinematic viscosity), IV (iodine value), CP (cloud point), PP (pour point), CFPP (cold filter plugging point), DV (density value), FP (flash point), SV (saponification value), and OSI (oxidation stability index). Many studies, such as, ref. 120–122 demonstrated that ML models can accurately estimate biodiesel properties using FAME composition or spectroscopic data (*e.g.*, NIRS). Among the various algorithms, multilayer perceptron neural networks (MLPNNs) are the most frequently used models in biodiesel research. In most cases, MLPNNs achieved very high predictive accuracy, often reporting *R*^2^ values above 0.95 and, in some studies, even approaching 0.999. These results indicate that MLPNNs can outperform traditional statistical approaches such as LR (linear regression) and RSM (response surface methodology).

In addition to property prediction, ML has been extensively applied to model FAME yield in enzymatic, alkaline, and acid-catalysed transesterification processes. In biodiesel modeling, process variables including methanol-to-oil ratio, catalyst concentration, reaction temperature, reaction time, enzyme loading, and water content have frequently been employed as model inputs. The reported results showed that machine-learning methods can successfully represent the complex nonlinear relationships between reaction conditions and biodiesel yield.^123–125^ Advanced hybrid models have also been introduced to improve performance. For example, ANFIS (Adaptive Neuro-Fuzzy Inference System),^126^ PSO-LSSVM (Particle Swarm Optimization – Least Squares Support Vector Machine),^127^ and MLPNN-GA (Genetic Algorithm-optimized neural network)^128^ demonstrated enhanced prediction capability. In particular, PSO-LSSVM showed slightly better accuracy than ANFIS in modeling biodiesel properties.

Existing ML studies primarily use operating conditions and feedstock characteristics as input variables, whereas catalyst physicochemical descriptors such as surface area, pore volume, acidity/basicity, metal loading, and dispersion remain largely unexplored. Integrating these catalyst-specific features into ML models represents a promising direction for developing predictive structure–property–performance relationships and accelerating catalyst discovery.

### 4.9 Integrated catalyst–machine learning framework for waste-derived carbon catalysts

The integration proposed in this review is organized as an evidence-to-decision loop rather than as two parallel bodies of literature. As illustrated in Fig. 16, the framework connects catalyst development, physicochemical characterization, feedstock and reaction conditions, catalytic performance, machine learning, and experimental validation. The first layer records catalyst-synthesis descriptors, including waste/biomass precursor, activation agent, carbonization or calcination profile, functionalization route, metal identity, and metal loading. The second layer contains physicochemical descriptors that govern catalytic behavior, including BET surface area, pore volume and pore-size distribution, acid/base functionality, metal phase or dispersion where reported, and recoverability. The third layer combines feedstock descriptors (*e.g.*, FFA and moisture) with process variables such as alcohol-to-oil ratio, catalyst loading, temperature, reaction time, and mixing conditions. The fourth layer defines engineering outputs, including FAME yield, biodiesel properties, catalyst stability, reuse-cycle performance, and metal leaching.

**Fig. 16:**
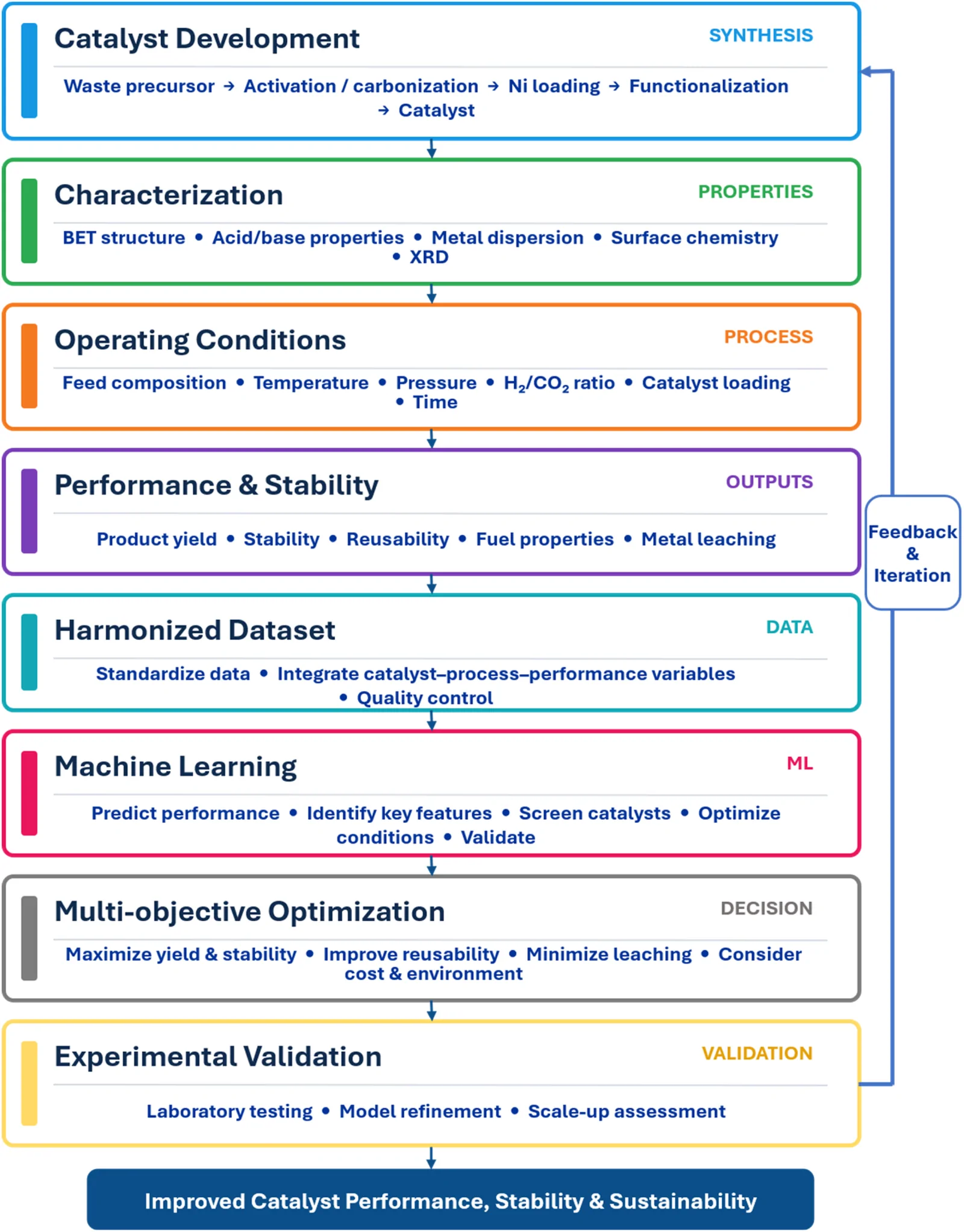
An integrated catalyst–machine learning framework linking catalyst development, process conditions, catalytic performance, and data-driven biodiesel optimization.

These interconnected descriptors form a harmonized catalyst–process–performance dataset, providing the basis for catalyst-aware ML analysis. Unlike process-only models that primarily rely on reaction and feedstock variables, catalyst-aware models can incorporate catalyst composition and physicochemical characteristics alongside operating conditions. This expanded input space can support prediction of catalytic performance, identification of influential descriptors, and screening of promising catalyst formulations and operating conditions. Model assessment should include independent validation where available, together with interpretable feature analysis and uncertainty-aware reporting.^102–104^

The resulting ML outputs can support decision-making and multi-objective optimization, where biodiesel yield is considered alongside catalyst stability, reusability, metal leaching, and sustainability. Promising catalyst formulations or operating windows identified through ML can then be prioritized for experimental validation. The resulting experimental data can be standardized and used for subsequent model updating, thereby establishing a closed evidence-to-decision loop between catalyst development, data-driven analysis, and experimental validation.

## 5. Research limitations and critical knowledge gaps

### 5.1 Experimental limitations in biodiesel production using metal-doped activated carbon catalysts

Despite the growing body of literature demonstrating enhanced catalytic activity of metal-doped AC systems, significant scientific and translational gaps remain unresolved.

#### 5.1.1 Lack of feedstock standardization and cross-comparability

Biodiesel feedstocks exhibit substantial variability in FAME composition, free fatty acid (FFA) content, and moisture level (*e.g.*, edible oils, non-edible oils, waste cooking oil, algal oils).^27,136^ Most experimental studies evaluate catalysts using a single feedstock under optimized laboratory conditions, limiting direct comparison across studies. The absence of standardized feedstock reporting frameworks constrains the development of universal structure–activity performance correlations for metal-doped AC systems.

#### 5.1.2 Insufficient long-term stability and leaching quantification

Although high initial biodiesel yields are frequently reported, systematic durability assessments remain limited. Reusability testing is often restricted to a few cycles, and quantitative analysis of active metal leaching (*e.g.*, Ca^2+^, transition metals) is inconsistently performed.^52,59–61,80^ Metal leaching may result in catalyst deactivation, biodiesel contamination, and additional purification requirements. Without standardized regeneration and leaching protocols, industrial feasibility cannot be reliably evaluated.

#### 5.1.3 Laboratory-scale bias and limited scale-up validation

The majority of studies are confined to small batch reactors operating under controlled conditions. Data on continuous-flow systems, catalyst ageing under realistic process streams, and long-term operational stability remain scarce.^34,56^ Furthermore, integrated techno-economic analysis (TEA) and life-cycle assessment (LCA) incorporating the energy demand of catalyst preparation (*e.g.*, calcination temperatures) and end-of-life considerations are limited.^56^ This gap restricts the reliable translation of laboratory findings to industrial applications.

#### 5.1.4 Unresolved pore-structure–mass-transfer trade-offs

Metal impregnation increases active site density but may reduce surface area, pore volume, and micropore accessibility.^59,80^ Given the steric limitations of triglyceride molecules, diffusion resistance may become rate-limiting in dense AC matrices. However, systematic mapping between pore architecture, metal dispersion, and catalytic activity remains insufficient, limiting rational optimization of metal loading strategies.

#### 5.1.5 Environmental trade-offs of metal doping strategies

Although waste-derived AC supports enhance circular economy potential, metal doping introduces additional material inputs and energy-intensive processing steps.^56,60^ Without a comprehensive life-cycle assessment, the net sustainability advantage of metal-doped AC catalysts relative to simpler waste-derived systems remains uncertain.

### 5.2 Methodological limitations in machine learning modeling of biodiesel production

ML has increasingly been applied to biodiesel yield prediction and process optimization; however, methodological inconsistencies and dataset limitations still exist as follows:

#### 5.2.1 Small, heterogeneous, and non-standardized datasets

Many ML studies rely on limited datasets derived from single laboratory campaigns. Feature engineering varies widely, including inconsistent physicochemical descriptors and FAME composition representations. Dataset fragmentation restricts generalization and increases the risk of overfitting.

#### 5.2.2 Overreliance on internal validation

High predictive accuracies are frequently reported based on internal cross-validation alone, with limited external validation using independent datasets or alternative feedstocks.^12,21,105^ This practice reduces confidence in model transferability and industrial robustness.

#### 5.2.3 Limited interpretability and uncertainty quantification

Most ML models prioritize predictive accuracy over interpretability. Few studies integrate uncertainty quantification, sensitivity analysis, or feature importance mapping.^105^ For catalyst screening and process control, transparent and uncertainty-aware predictions are essential.

### 5.3 Critical research gap: machine learning for metal-doped AC catalyst systems

Although ML has shown strong predictive performance in biodiesel modeling, its application to systems using metal-doped activated-carbon catalysts remains limited. Most existing studies rely mainly on feedstock and process variables, while detailed catalyst descriptors such as surface area, pore characteristics, metal identity and loading, and acid/base functionality remain underrepresented.

This limitation, together with the lack of standardized catalyst-specific datasets, restricts transferable catalyst screening and rational design. Robust ML frameworks therefore require harmonized datasets linking catalyst composition and physicochemical properties with feedstock characteristics, reaction conditions, catalytic performance, stability, reusability, and leaching data.^21,105^ Such datasets can support transferable structure–property–process–performance learning and experimentally validated catalyst optimization. The existing limitations and technical bottlenecks of current catalyst methodologies are systematically outlined in Fig. 17.

**Fig. 17:**
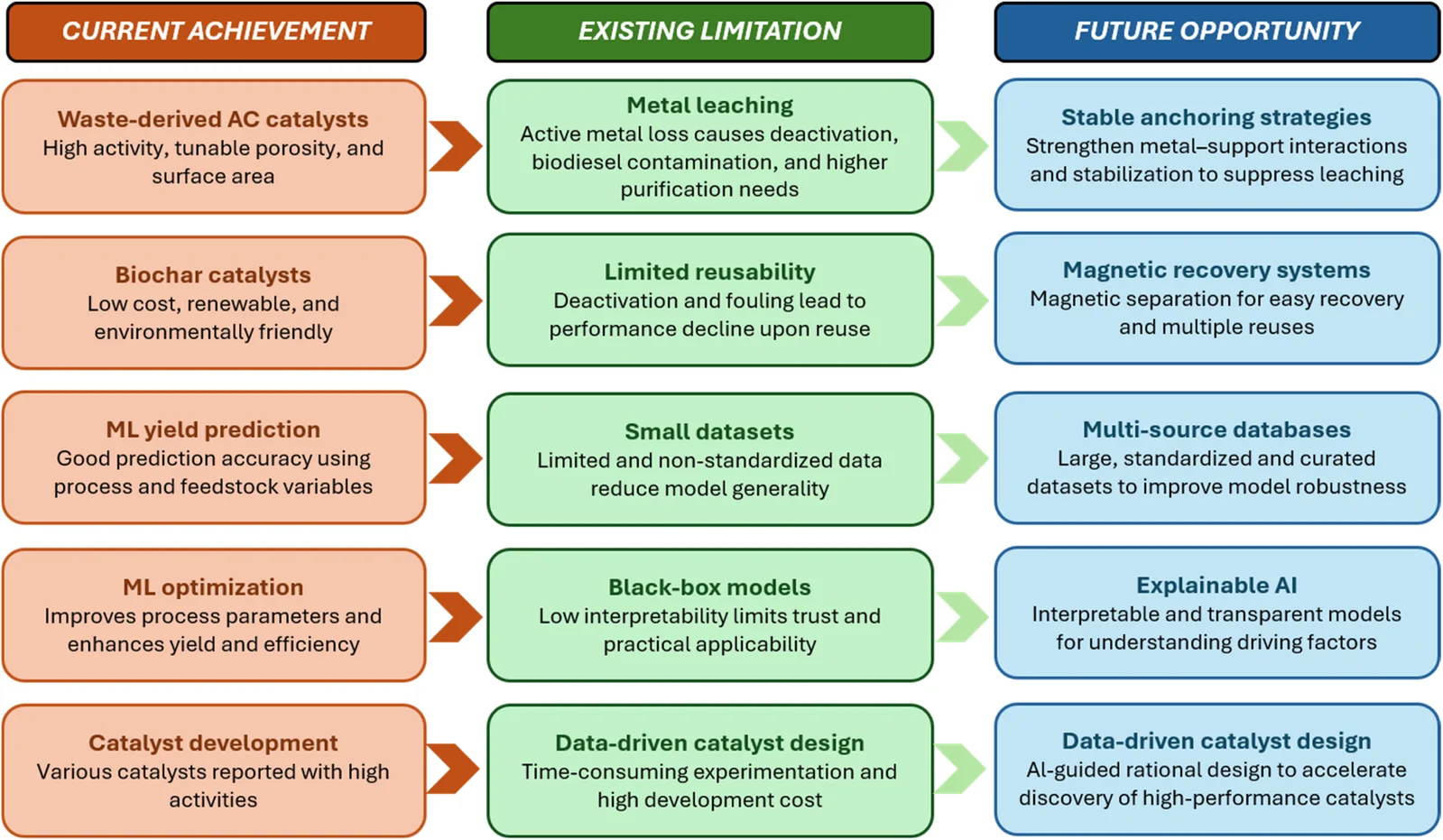
Visual mapping of current achievements, principal limitations, and corresponding future opportunities for waste-derived carbon-catalyst and ML development.

## 6. Future work directions

Reaching a threshold of reliable, sustainable, and industrially relevant biodiesel production using metal-doped AC catalysts requires addressing the critical gaps mapped out in Fig. 17. To realize this strategic roadmap, the following future research pathways are especially promising:

### 6.1 Standardized, open datasets for ML-enabled catalyst discovery

Creation of shared, well-annotated datasets is essential. This includes integrating catalyst synthesis parameters (precursor, activation agent, calcination profile, metal loading), textural/chemical characterization (BET, XRD, XPS, FTIR), reaction conditions, yields, leaching data, and downstream fuel properties. Standardization will enable reproducible benchmarking, foster transfer learning across feedstocks, and support robust uncertainty quantification and external validation of ML models.^12,21^

### 6.2 ML-guided multi-objective optimization for catalyst formulation

Employing interpretable and multi-objective ML frameworks to navigate trade-offs among activity, stability, porosity, and environmental metrics is crucial. Hybrid workflows that combine physics-based constraints (reaction thermodynamics/kinetics) with data-driven surrogates (PINNs or surrogate ensembles) can speed up experimental campaigns while ensuring physically consistent predictions.^105,111^

### 6.3 Incorporate leaching and lifecycle objectives into optimization targets

Expanding optimization objectives to explicitly minimize metal leaching and the embodied environmental impacts of catalyst preparation (energy for activation, metal consumption), enabling selection of dopant loadings and activation protocols that balance catalyst performance with sustainability. Integrated techno-economic and LCA analyses should accompany catalyst proposals to lower barriers to scale-up.^60,67^

### 6.4 Design of bifunctional and magnetic catalysts for one-pot processing and recovery

Prioritizing the development of bifunctional AC catalysts (combined acidic and basic sites) and magnetic composites (*e.g.*, Fe-based inclusions) to enable simultaneous esterification/transesterification of high-FFA feedstocks and facile recovery *via* magnetic separation. ML can help identify synthesis windows that maximize bifunctionality while preserving pore structure and minimizing agglomeration.^59^

### 6.5 Robust reusability protocols and regeneration strategies

Systematically investigating regeneration methods (thermal and chemical reactivation) and passivation-resistant dopants that reduce pore blockage and agglomeration. Standardized reuse-test protocols should be encouraged, including the number of cycles, leaching assays, and post-reuse characterization, to build comparative longevity benchmarks.^67^

## 7. Conclusion

This review highlights the strong potential of waste-derived metal-doped activated carbon and biochar catalysts for sustainable biodiesel production. Their tunable surface chemistry, pore structure, and acid–base properties can provide high catalytic activity while supporting catalyst recovery and reuse. However, catalyst performance varies with precursor characteristics, activation and functionalization strategies, active-phase composition, feedstock, and reaction conditions, making direct comparisons difficult. Catalyst stability, metal leaching, deactivation, regeneration, and scale-up remain important challenges.

Machine learning is increasingly used for biodiesel yield prediction, process optimization, and fuel–property estimation. However, most existing models rely mainly on feedstock and operating variables, with limited incorporation of catalyst-specific descriptors such as surface area, pore characteristics, metal loading, and acid/base functionality. This limits their application to transferable catalyst screening and design.

Future progress requires integrating catalyst engineering and machine learning within a unified structure–property–process–performance framework. Key priorities include standardized catalyst–process–performance datasets, catalyst-aware and interpretable ML models, systematic durability, regeneration, and leaching studies, and experimental validation of ML-guided catalyst screening and optimization. Such integration can move ML beyond process-only prediction toward more effective catalyst-aware design and optimization, supporting the development of stable, reusable, and scalable waste-derived carbon catalysts for sustainable biodiesel production.

## Author contributions

Menna Ebrahim: conceptualization, data curation, formal analysis, investigation, methodology, software, validation, visualization, writing – original draft, and writing – review & editing. Mostafa Mahmoud: data curation, investigation, methodology, software, validation, visualization, and writing – review & editing. Fatma H. Ashour: conceptualization, formal analysis, funding acquisition, investigation, project administration, resources, supervision, validation, and writing – review & editing. Osama A. Ibrahim: data curation, investigation, methodology, software, supervision, validation, visualization, and writing – review & editing. Nourhan H. Khashaba: conceptualization, data curation, investigation, methodology, resources, supervision, validation, visualization, and writing – review & editing.

## Conflicts of interest

The authors state that they have no financial conflicts of interest or personal relationships that could have influenced the research presented in this paper.

## Supplementary Material

RA-OLF-D6RA05871J-s001

## Data Availability

No new data were generated in this review. The article is based on previously published literature, and all data supporting the findings are available in the cited references. Supplementary information (SI) is available. See DOI: https://doi.org/10.1039/d6ra05871j.
