# Supplementary material for: Bridging waste-derived carbon catalyst innovation and machine learning for sustainable biodiesel production: a comprehensive review
Source: RSC Adv. 2026 Oct 19;16(48):63509–41. doi: 10.1039/d6ra05871j (PMC13678681; doi:10.1039/d6ra05871j)
Supplement: Supplementary file 1 [file RA-016-D6RA05871J-s001.pdf]

Supplementary Information (SI) for RSC Advances.
This journal is © The Royal Society of Chemistry

Supplementary Information

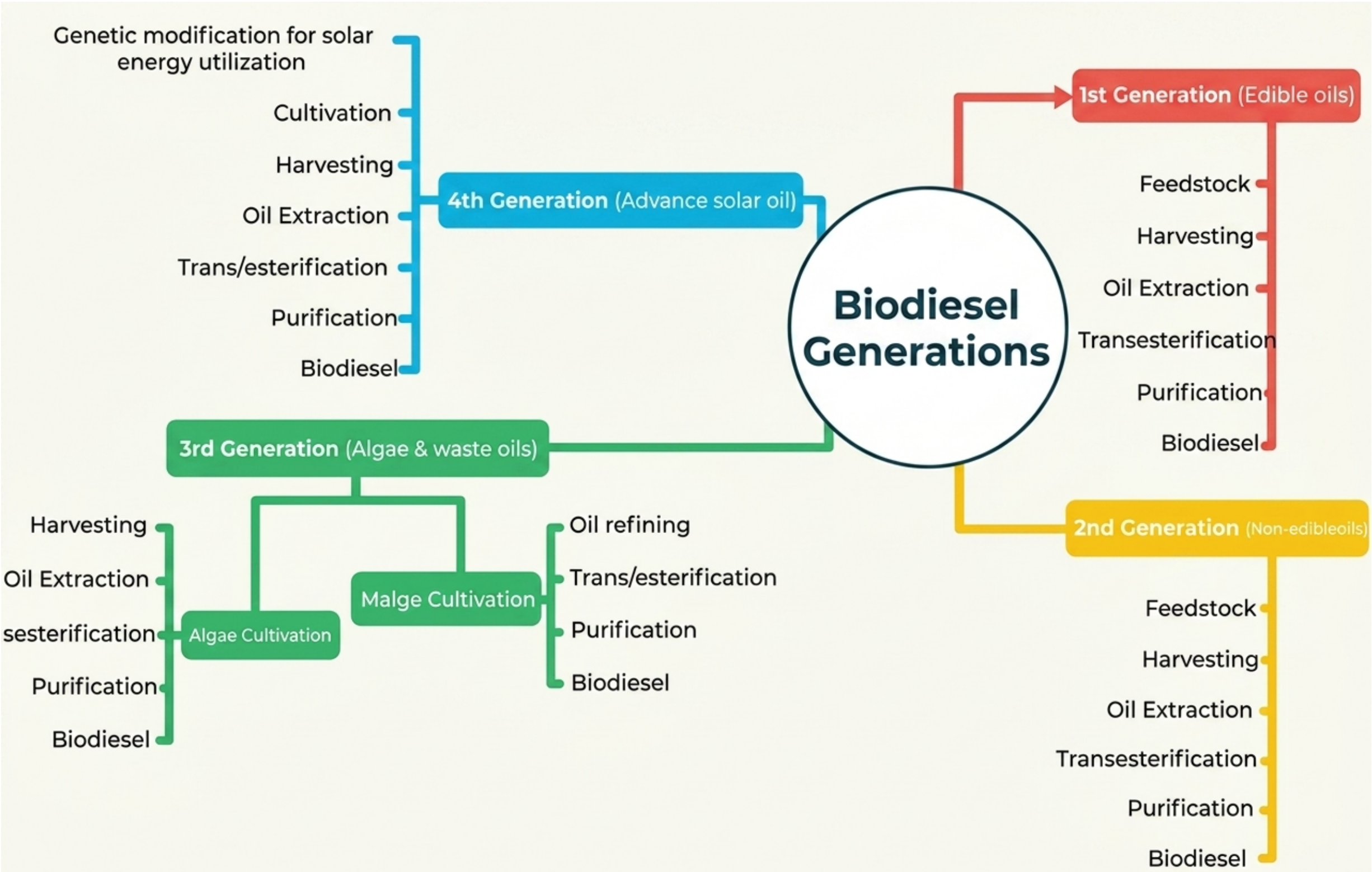

Fig. S1. Various categories of feedstocks across biodiesel production generations.

Table S1: Reported optimal operating conditions and corresponding biodiesel yields.

Feedstock	Catalyst / Material	Catalyst Type	Reaction Time (min)	Temp (°C)	Alcohol: Oil Ratio	Catalyst Loading	Yield (wt.%)	Ref.
Scenedesmus obliquus lipid	H_2SO_4	Homogeneous acid	240	60	30:1	10 wt%	96.7	[83]
Jatropha oil	KOH	Homogeneous base	120	45	9:1	2 wt%	90–95	[46]
Karanja oil	KOH	Homogeneous base	30	55	9:1	0.5 wt%	80–85	[46]
Corn oil	KOH	Homogeneous base	60	80	9:1	2 wt%	85–96	[46]
Honne oil	KOH	Homogeneous base	120	60	8:1	1.25 wt%	89	[50]
Waste cooking oil	KOH	Homogeneous base	1	60	9:1	1 wt%	96.3	[47]
Chrysophyllum oil	KOH	Homogeneous base	40	65	9:1	1 wt%	99.2	[29]
Waste cooking oil	KOH	Homogeneous base	70	60	3:1	1 wt%	94.0	[49]
Sea mango oil	Sulfated zirconia/Al_2O_3	Heterogeneous acid	180	180	8:1	6 wt%	83.8	[48]
Soybean oil	Sulfated ZnO	Heterogeneous acid	120	65	6:1	4 wt%	80.2	[48]

Feedstock	Catalyst / Material	Catalyst Type	Reaction Time (min)	Temp (°C)	Alcohol: Oil Ratio	Catalyst Loading	Yield (wt.%)	Ref.
Palm oil	Zirconia/AC–zeolite	Heterogeneous acid	120	65	10:1	10 wt%	84.2	[48]

Table S2: Physicochemical properties of biodiesel produced from different feedstocks and catalyst systems.

Feedstock / Catalyst	Viscosity @ 40 °C (mm^2/s)	Density	Flash Point	CFPP (°C)	Heating Value	Ester Content (%)	Cetane Number	Pour Point (°C)	Feedstock / Catalyst	Reference
**Hazelnut Oil + NaOH**	4.128	873.06 kg/m^3 (15 °C)	177 °C	–6	39,883 kJ/kg	–	–	–	Hazelnut Oil + NaOH	[52]
**Rapeseed Oil + NaOH**	4.5	0.86 (298 K)	409 K	–	38.8 MJ/L	99.4	–	–	Rapeseed Oil + NaOH	[35]
**Rapeseed Oil + KOH**	4.5	0.88 (298 K)	410 K	–	38.7 MJ/L	99.4	–	–	Rapeseed Oil + KOH	[35]
**ESG Oil + Heteropoly Acid**	5.0	0.879 (15 °C)	127 °C	−3	38.67 MJ/kg	–	49	−3	ESG Oil + Heteropoly Acid	[36]
**Soybean Oil + Heteropoly Acid**	4.08	0.885 (15 °C)	69 °C	−15	39.75 MJ/kg	–	48.2	−15	Soybean Oil + Heteropoly Acid	[36]
**Cottonseed Oil + Heteropoly Acid**	4.0	0.874 (15 °C)	70 °C	−3	40.32 MJ/kg	–	–	−3	Cottonseed Oil + Heteropoly Acid	[36]
